# A meta-analysis of mannose-binding lectin gene polymorphisms with the risk of recurrent vulvovaginal infections

**DOI:** 10.1038/s41598-020-63261-8

**Published:** 2020-04-08

**Authors:** Namarta Kalia, Jatinder Singh, Akash Kumar Rauniyar, Manpreet Kaur

**Affiliations:** 1 Department of Molecular Biology & Biochemistry, Guru Nanak Dev University, Amritsar, India; 2 Department of Information Technology, University of Turku, Turku, Finland; 3 Department of Human Genetics, Guru Nanak Dev University, Amritsar, India

**Keywords:** Genetics, Immunology, Biomarkers, Diseases, Pathogenesis, Rheumatology, Risk factors

## Abstract

The genetic variants of Mannose-Binding Lectin, a vital component of innate immunity have been studied with acute/recurrent vaginal infections ((R)VVI) and presented inconclusive findings. Therefore, a systematic review and meta-analysis of published data were conducted to assess the possible role of these variations in (R)VVI. A comprehensive search was made using PubMed, Web of Science and Google scholar till June 18, 2019. A total of 12 studies met the specified criteria and were included in the analysis. Different comparisons were made on the basis of the outcome of interest that resulted in the filtering of studies for the pooled analysis to find an association using the standard genetic models. Odds ratio (OR) with 95% confidence interval (CI) was chosen as the effect measure for the data synthesis. The trim and fill technique was applied to adjust the publication bias. The meta-analysis revealed the significant association (p < 0.05) of rs1800450 polymorphism with RVVI risk (OR ≥ 3.5) in all the genetic models. The subgroup analysis identified the same association in Caucasian and Mixed ethnicity. Quantitative synthesis based on RVVC showed>3.5 fold risk of disease development accredited to rs1800450. A combined evaluation of Exon1 variants showed no association with (R)VVI. This meta-analysis suggests rs1800450 polymorphism as a genetic predisposing factor for RVVI, but to reinforce, further studies with a larger sample size are warranted.

## Introduction

Vulvovaginal infections (VVI) account for a huge fraction of gynaecological outpatient visits by woman of childbearing age and include about 10 to 20% of consultations only. Bacterial vaginosis (BV), vulvovaginal candidiasis (VVC) and trichomoniasis (TV) are the frequently encountered VVI in clinical practice^1^. However, the important issues are their high recurrence rates (RVVI) and the associated severe pregnancy outcomes as well as infectious diseases^2^. High proportion of opportunistic pathogens, which are either normally dwelling or transmitted sexually have been suggested as the reason behind the development of VVI and RVVI, collectively abbreviated in this study as (R)VVI^2^. Nevertheless, universal presence of asymptomatic cases implies that the shared risk factors of (R)VVI including excessive sexual activity, antibiotics/contraceptives use and ethnicity are simply responsible for increasing the vaginal colonisation of potentially dangerous microbial species but not disease^3^. Hence, the presence of symptomatic/asymptomatic (R)VVI cases are mainly accredited to the differences in women’s immunity, conferred partially or wholly by genetic variations^1^. Therefore, genetic exploration of immune mediators that ultimately decides the host susceptibility to symptomatic (R)VVI is necessary.

One such important mediator is human Mannose-Binding Lectin (MBL), which is a vital component of systemic as well as mucosal innate immunity^4^. It’s a multimeric soluble protein encoded by *MBL2* mapped to 10q21.1. Evidences have been provided regarding MBL binding to the molecular patterns of (R)VVI pathogens and thus its important role in first line of vaginal defense ranging from direct opsonophagocytosis, activation of C system with subsequent indirect opsonisation, phagocytosis, ROS production and pro-inflammatory signaling^5–7^. Moreover, MBL levels has been assessed in vaginal fluid/serum of (R)VVI cases that has suggested its effective role in acute phase defense, although defective MBL production attributed to genetic variations appears to be predisposing women to (R)VVI^8–12^.

Several genetic variations of *MBL2* have been suggested to have potential functional consequences on encoded protein and its levels, even though only six of them are functionally recognised and widely studied^13^. Three variant alleles in Exon 1 of *MBL2* including T(also known as D allele), A(also known as B allele) and A(also known as C allele) of rs5030737 (codon 52 and C > T transition), rs1800450 (codon 54 and G > A transition) and rs1800451 (codon 57 and G > A transition) single point mutations respectively, often pooled together as “O” owing to their similar effect in the collagenous region of MBL monomers that results in non-functional high order oligomers, while their wild type alleles (C, G and G respectively) are referred as “A”^14^. These variants together with three promoter variants including rs11003125 (a C > G transition also known as L > H variant), rs7096206 (a G > C transition also known as Y > X variant) and rs7095891 (C > T transition also known as P > Q variant), the other well-known protein expression regulators, form haplotypes including HYPA, LYPA, LYQA, LYPB, LXPA, LYQC and HYPD, which have been documented to alter soluble MBL levels by interfering in its transcription as well as translation^15^. Therefore, analysis of *MBL2* genetic variations could be informative in identifying the women at risk of developing (R)VVI and to validate the therapeutic possibility of emerging MBL substitution therapy for this condition.

Thus, by considering the importance of gene in view, studies have examined the possible association between *MBL2* polymorphisms in susceptibility to (R)VVI in different populations^8–10,16–24^. Nevertheless, owing to ethnic diversity and underpowered study design, the conclusions provided by these reports are still controversial, demanding further in-depth investigation to establish the relationship between the two. Therefore, in order to provide scientifically rigorous summarised information, a systematic review of hitherto literary evidences and the quantitative synthesis of data thus obtained, were performed in this study by applying a well recognised statistical method—a meta-analysis, making the current investigation a primary approach towards it.

## Methodology

### Outcome of interest

The outcomes of interest were women, who have experienced either at least one episode of any of the common vaginal infections (VVI) or its repeated episodes (RVVI) as per the original definitions. For VVI, the standard clinical symptoms and laboratory based diagnosis positive for BV, VVC and TV should be in consistent with European (IUSTI/WHO) guidelines on vaginal discharge management^25^. Whereas, the condition of RVVI is defined as the repetition rates as high as 30–50% within 3 months for recurrent BV (RBV), while ≥4 repetitive episodes of VVC in 12 months for recurrent VVC (RVVC)^2^. Similarly, cases of recurring TV (RTV) should have recurrence rates as high as 5–8% within 2 months of initial diagnosis^26^. VVI or RVVI denoted as (R)VVI are the primary study outcome, while different categories (R)BV, (R)VVC and (R)TV are the secondary study outcome in the present study.

### Inclusion and exclusion criteria

The study was performed in line with the preferred reporting items for systematic reviews and meta-analyses (PRISMA) guidelines^27^. All those retrieved studies were incorporated that met the following criteria: (i) it must have assessed an association between *MBL2* gene polymorphisms and (R)VVI [At least any one of (R)BV, (R)VVC, (R)TV or more than one or all] susceptibility; (ii) conducted under a case-control design; (iii) had a clear description of (R)VVI cases (as aforementioned) and controls (women who never assessed positive for symptomatic VVI nor complaint of its recurrent episodes; (iv) must have an accessible genotype distribution in cases and controls; (v) must be published in English language or have an English abstract; (vi) Data collected and analysis performed should be valid from statistical point of view.

The studies were excluded if: (i) they did not have an accessible genotype information; (ii) involved evaluation of MBL protein levels or vaginal microbiota distribution only, under a case-control design; (iii) case only studies without a comparison group; (iv) functional studies involving *in-vivo*/*in-vitro* analysis; (v) duplicates; (vi) review articles, comments or animal studies.

### Literature search

The electronic databases including PubMed (MEDLINE), Web of Science and Google scholar were used to retrieve articles that have studied an association between the *MBL2* polymorphisms and risk of (R)VVI. The combination of terms used were: “MBL” OR “*MBL2*” OR “mannose binding lectin” OR “mannose-binding lectin” OR “mannose binding lectin 2” OR “mannose-binding lectin 2” AND (”vaginal infections” OR “cervicovaginal infections” OR “Recurrent vulvovaginal infections” OR “RVVI” OR “VVI” OR “vulvovaginal candidiasis” OR “Recurrent Vulvovaginal Candidiasis” OR “bacterial vaginosis” OR “Recurrent bacterial vaginosis” OR “Trichomoniasis” OR “Recurrent Trichomoniasis”). We searched the electronic databases from inception to June 18, 2019 without any restrictions. The reference lists of resulting articles were also screened to find out the relevant publications that could have been missed in the initial search. Moreover, the cited literature in review articles was manually scanned for possible studies.

### Data extraction and quality assessment

By stringently following the inclusion/exclusion criteria, an independent extraction of data from the selected studies was performed by three investigators (NK, AKR and MK) on a customised data extraction e-form (MS Excel). For each study, the following information was collected: first author, year and country of origin, ethnicity, study type, sample size, age distribution of cases and controls, polymorphisms detected, genotyping methods, genotypes distribution for each polymorphism in both cases and controls, and association status with (R)VVI. Furthermore, the quality of selected studies were independently evaluated by two reviewers (NK and MK) using star-rating system (from 0–9) of Newcastle-Ottawa Scale (NOS) for quality assessment^28^. Those studies that scored ≥5 stars were ranked from moderate to higher methodological quality. Contradictions in regard to data extraction, study eligibility and quality assessment were resolved by systematic discussion in the presence of third reviewer (JS).

### Data synthesis

Odds ratio (OR) with 95% confidence interval (CI) was chosen as the effect measure for the quantitative synthesis conducted on the basis of combination formed from different (R)VVI and *MBL2* polymorphisms. The OR-estimates were pooled based on allelic, homozygote, heterozygote, dominant and recessive genetic models for each comparison. χ^2^–based Cochran’s Q statistics were used to assess heterogeneity between studies, which signifies its presence at p-value < 0.05. To further quantify the amount of variability between studies, I^2^ index was used to represent the presence of low, moderate and high inconsistency with increasing value of I^2^ index *i.e*. < 25%, 50% and >75% respectively^29^. To account for observed significant (p < 0.05) heterogeneity among studies, the random-effects model was applied. However, in case of no heterogeneity (p > 0.05) a fixed-effects model was used for pooling results. Moreover, the publication bias was assessed by visually interpreting the funnel plots and the quantification of it was performed by the Egger’s linear regression test and Begg & Mazumdar’s rank correlation test, wherein the p < 0.05 depicts statistically significant publication bias^30^. Further, to adjust the observed publication bias, trim and fill technique was used for recalculating the effect size (ES) of the genetic models^31^. Sensitivity analyses was also carried out to evaluate the contribution of each study to the pooled OR estimates by sequentially removing one study at a time and re-computing the summary ES for the rest of the outcomes. Distinct ethnicities were considered as moderators and the pooled results were stratified and compared among subgroups of Asian, Caucasian, Egyptian and Mixed population formed because of it. The countries of Latin America, particularly Brazil, Mexico and Colombia have widespread racial mixing (made up of people of several different origins from the original natives to Portuguese, European, Black African, Japanese and Arab colonists), hence generally regarded as of Mixed ethnicities. All these statistical analysis was performed by ProMeta software v 3.0 (Internovi, Cesena FC, Italy). Hardy-Weinberg equilibrium (HWE) in the controls was determined using χ^2^ test. The adequate sample size needed to achieve the statistical power of each *MBL2* polymorphism was calculated by genetic association study power calculator (http://csg.sph.umich.edu/abecasis/gas_power_calculator/) using global minor allele frequency of each polymorphism. Bonferroni’s correction was applied to control inflation of the type I error rate. p < 0.05 was considered statistically significant.

## Results

### Characteristics and quality of included studies

The preliminary search resulted in 187 studies including 23 from PubMed (MEDLINE), 143 from Google Scholar and 21 from Web of Science core collection. However, only 16 studies were found potentially eligible after careful evaluation of titles/abstracts and removal of duplicate studies. Finally, only 12 studies^8–10,16–24^ met the specified inclusion and exclusion criteria and thus incorporated in the present study (PRISMA flow chart shown in Fig. S1). Moreover, the follow up search after June 18, 2019 did not yield any relevant study for inclusion. The main characteristics of each included study are summarized in Table 1. The genotype and allelic distributions of all the polymorphisms except those reported by single study only, are shown in Table S1. The genotype distributions in controls were in accordance with HWE in all the studies except two^17,22^. The systematic quality assessment of each included study following the NOS criteria is illustrated in Table S2. The average NOS stars scored by the included studies was 7.5 (range 7–8), indicating high methodological quality of the included studies.

**Table 1:** Characteristics of included studies.

First Author, Year [Ref]	Country	Ethnicity	Type of study	Total Participants		Cases			Controls		Genotyping Methods	SNPs Detected	Association Status
				n	Age, years mean ± S.D (range)	Types	n	Age, years mean ± S.D (range)	n	Age, years mean ± S.D (range)			
Babula *et al*. 2003^8^	Latvia	Caucasian	HB	—	—	RVVC	42	26.8 (18–35)	43	25.4 (18–35)	PCR-RFLP	rs1800450	Risk associated
Liu *et al*. 2006^9^	China	Asian	HB	—	—	VVC/RVVC	51/6	32.86 ± 8.07/29.17 ± 6.05	54	30.87 ± 5.39	PCR-RFLP	rs1800450	Risk associated
De Seta *et al*. 2007^16^	Italy	Caucasian	HB	201	30 (18–40)	RBV	71	—	130	—	MTA	EXON 1 SNPs (rs5030737, rs1800450, and rs1800451) Combined effect	No association
Girlado *et al*. 2007^17^†	Brazil	Mixed	HB	177	—	VVC (acute + recurrent)/BV (acute + recurrent)	78 (28 + 50)/33 (13 + 20)	31.5 (18–68)/33.0 (18–57)	66	33.5 (16–70)	PCR-RFLP	rs1800450 and rs1800451	rs1800450 associated with risk of both RVVC and RBV, rs1800451 (No association)
Donders *et al*. 2008^18^	Belgium	Caucasian	HB	—	—	RVVC	109	36 ± 9.27	55	33.3 ± 10.4	PCR-RFLP	rs1800450	Risk associated
Milanese *et al*. 2008^10^	Italy	Caucasian	HB	322	30 (16–47)	RVVC/RBV	85/74	—	163	—	MTA	EXON 1 SNPs (rs5030737, rs1800450, and rs1800451) Combined effect	No association
Wojitani *et al*. 2012^19^†	Brazil	Mixed	HB	—	—	RVVC	100	32.4 (18–50)	100	29.9 (19–49)	PCR-RFLP	rs1800450	Risk associated
Velazquez-Hernandez *et al*. 2017^20^	Mexico	Mixed	HB	354	36.4 ± 10.3 (17–67)	CVI (BV + VVC + TV)	128 (72 + 49 + 7)	—	226	—	qPCR	rs1800450 and rs1800451	No association
Hammad *et al*. 2018^21^	Egypt	Egyptian	HB	—	—	RVVC	59	(30–40)	59	(30–40)	PCR-RFLP	rs1800450	Risk associated
Kalia *et al*. 2017^22^	Indian	Asian	HB	—	—	RVVI (BV + VVC + MI)	258 (97 + 62 + 41)	29.33 ± 8.32	203	9.33 ± 8.17	ARMS-PCR	rs11003125, rs7096206, rs7095891, and rs1800450	rs7096206 risk associated, Other SNPs (No association)
Kalia et al., 2019^23^	Indian	Asian	HB	—	—	RVVI (BV + VVC + MI)	109 (56 + 28 + 25)	29.22 ± 7.95	109	29.42 ± 7.70	Sanger Sequencing	rs10824792, rs2120132, rs2120131, rs2165813, rs2099903 and rs2099902	rs10824792 risk associated, Other SNPs (No association)
Kalia *et al*. 2019b^24^	Indian	Asian	HB	—	—	RVVI (BV + VVC + MI)	258 (97 + 62 + 41)	29.33 ± 8.32	203	9.33 ± 8.17	PCR-RFLP	rs11003124, rs7084554, rs36014597 and rs11003123	rs7084554 and rs36014597 risk associated, Other SNPs (No association)

**Notes:** †Age is given as median (range), “—” not reported, Synonyms of well known *MBL2* secretor polymorphisms including Exon 1 SNPs are rs5030737 (Codon 52, C > T transition, A/D), rs1800450 (Codon 54, G > A transition, A/B), rs1800451 (Codon 57, G > A transition, A/C) and promoter polymorphisms are rs11003125 (C > G transition, L/H), rs7096206 (G > C transition, Y/X), rs7095891 (C > T transition, P/Q).

**Abbreviations:** HB = Hospital based, VVC = vulvovaginal candidiasis, RVVC = Recurrent vulvovaginal candidiasis, RBV = Recurrent Bacterial Vaginosis, BV = Bacterial Vaginosis, CVI = Cervicovaginal infections, MI = Mixed Infections RVVI = recurrent vulvovaginal infection, TV = trichomoniasis, PCR-RFLP = polymerase chain reaction restriction fragment length polymorphism, MTA = Melting temperature assay, qPCR = real-time polymerase chain reaction with Taqman probes, ARMS-PCR = Amplification refractory mutation system-PCR, SNPs = single nucleotide polymorphisms.

### Association between MBL2 rs1800450 polymorphism and RVVI

**Figure 1:**
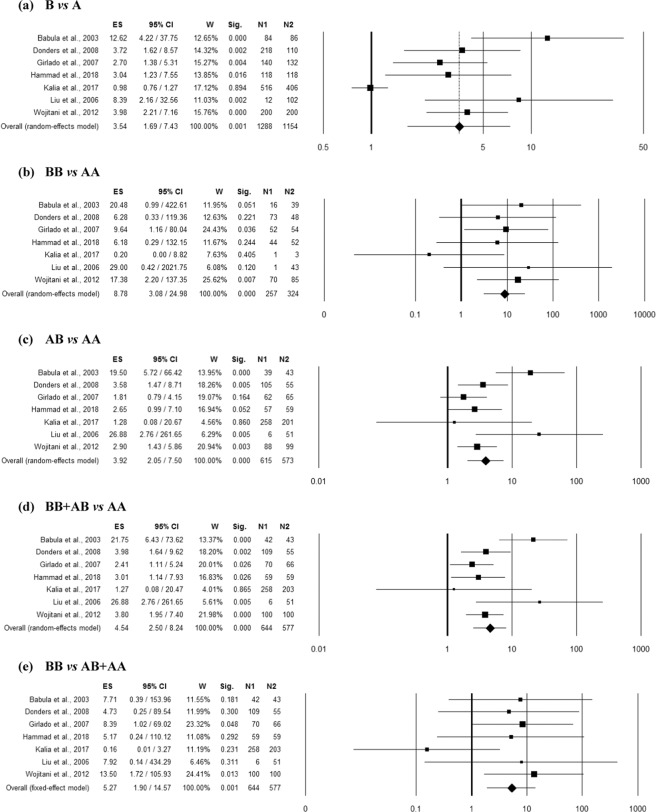
Forest plots for the association between rs1800450 (A/B = G/A allele) polymorphism and RVVI under the (**a**) allelic, (**b**) homozygote, (**c**) heterozygote, (**d**) dominant and (**e**) recessive mode of inheritance for the studies including Babula *et al*.^8^; Donders *et al*.^18^; Girlado*et al*.^17^; Hammad *et al*.^21^; Kalia *et al*.^22^; Liu *et al*.^9^ and Wojitani *et al*.^19^. Black boxes represents the value of odds ratio (OR) and the size of the boxes is inversely proportional to the size of the result study variance, so that more precise studies have larger boxes. Horizontal line is the 95% Confidence Interval (CI) of OR. The summary OR is represented by the diamond, where the center of the diamond indicates the OR. The ES is effect size expressed as OR; W, weight; Sig, statistical significance; N1, cases; N2, controls.

Seven studies^8,9,17–19,21,22^ provided sufficient data (comprising 577 controls and 644 RVVI cases) for the pooled analysis to find an association. Homogeneity between studies was observed in homozygous (BB *vs* AA) and recessive (BB vs BA + AA) genetic models (Table S3). However, high inconsistency between studies was observed in allelic contrast (B vs A) and moderate inconsistency in heterozygous (BA vs AA) as well as dominant (BB + BA vs AA) genetic models. The overall ES indicated significant association of *MBL2* rs1800450 polymorphism with RVVI risk in all the genetic models including allelic contrast (p = 0.001; Odds ratio (OR) = 3.54; 95% confidence interval (CI) = 1.69–7.43), homozygous (p < 0.0001; OR = 8.78; 95% CI = 3.08–24.98), heterozygous (p < 0.0001; OR = 3.92; 95% CI = 2.05–7.50), dominant (p < 0.0001; OR = 4.54; 95% CI = 2.50–8.24) and recessive (p = 0.001; OR = 5.27; 95% CI = 1.90–14.57) (Fig. 1). Funnel plot showed asymmetry (Fig. S2), with an effect on the ES as indicated by the trim and fill analysis (Table S4) in homozygous (estimated ES: OR = 8.12 [2.94–22.43], p < 0.0001; number of trimmed studies: 1) and recessive (estimated ES: OR = 2.76 [1.19–6.37], p = 0.018; number of trimmed studies: 2) genetic models. The Egger’s linear regression test confirmed the possible presence of a publication bias (p = 0.002) only in allelic contrast model (Table S3). However, the overall significant risk effect observed in the recessive mode of inheritance was lost (p = 0.09) after correction for multiple comparisons.

Moderator and Subgroup analysis: Further for the better interpretation of quantitative synthesis, we studied the effect of ethnicity on ES and variability among the included studies (Table S5). Two case-control studies were found eligible for the analysis in each ethnic group including Asian, Caucasian and Mixed population, while only one study belongs to Egyptian population (Fig. S3). The ES did not change significantly based on the ethnicities of the included studies in any genetic model. We re-accessed the possible link of *MBL2* rs1800450 polymorphism with RVVI in different ethnic groups (Table S5). The subgroup analysis identified the association *MBL2* rs1800450 polymorphism with increased risk of RVVI in Caucasian ethnicity in overall allelic contrast (p = 0.002; OR = 6.49; 95% CI = 1.97–21.38), homozygous (p = 0.025; OR = 11.16; 95% CI = 1.35–92.10), heterozygous (p = 0.015; OR = 7.91; 95% CI = 1.51–41.53) and dominant (p = 0.010; OR = 8.81; 95% CI = 1.67–46.43) genetic models. However, the observed significant risk effect was lost in the homozygous (p = 0.1) and heterozygous (p = 0.06) genetic models, when correction for multiple testing was applied. Moreover, high inconsistency was also found in heterozygous and dominant genetic models for Caucasian population. Additionally, Mixed ethnic group with 2 successfully included case-control studies showed an association with RVVI risk in all the genetic models including allelic contrast (p = 0.00001; OR = 3.37; 95% CI = 2.16–5.25), homozygous (p = 0.001; OR = 3.03; 95% CI = 2.94–57.19), heterozygous (p = 0.002; OR = 2.38; 95% CI = 1.39–4.07), dominant (p = 0.00001; OR = 3.13; 95% CI = 1.89–5.19) and recessive (p = 0.002; OR = 10.70; 95% CI = 2.45–46.68) even after Bonferroni correction with no observed heterogeneity at all. However, no significant (p > 0.05) association of rs1800450 polymorphism with RVVI susceptibility was found for Asian ethnicity in any genetic models (Table S5).

### Association between MBL2 rs1800450 polymorphism and RVVC

**Figure 2:**
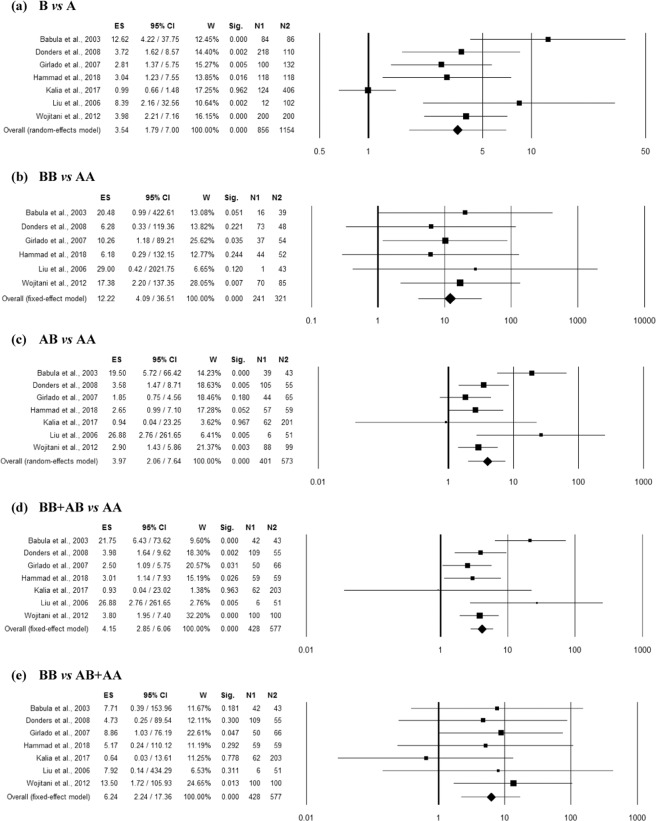
Forest plots for the association between rs1800450 (A/B = G/A allele) polymorphism and RVVC under the (**a**) allelic, (**b**) homozygote, (**c**) heterozygote, (**d**) dominant and (e) recessive mode of inheritance for the studies including Babula *et al*.^8^; Donders *et al*.^18^; Girlado *et al*.^17^; Hammad *et al*.^21^; Kalia *et al*.^22^; Liu *et al*.^9^ and Wojitani *et al*.^19^. Black boxes represents the value of odds ratio (OR) and the size of the boxes is inversely proportional to the size of the result study variance, so that more precise studieshave larger boxes. Horizontal line is the 95% Confidence Interval (CI) of OR. The summary OR is represented by the diamond, where the center of the diamond indicates the OR. The ES is effect size expressed as OR; W, weight; Sig, statistical significance; N1, cases; N2, controls.

Seven studies^8,9,17–19,21,22^ reported enough data (including 577 controls and 428 RVVC cases) for the pooled analysis of *MBL2* rs1800450 polymorphism and RVVC risk. Homogeneity between studies was observed in homozygous and recessive genetic models (Table S6). However, high inconsistency between studies was observed in allelic contrast and moderate inconsistency in heterozygous as well as dominant genetic models (Table S6). The overall ES indicated significant association of rs1800450 polymorphism with RVVC risk in all the genetic models including allelic contrast (p = 0.0002; OR = 3.54; 95% CI = 1.79–7.00), homozygous (p < 0.001; OR = 12.22; 95% CI = 4.09–36.51), heterozygous (p < 0.001; OR = 3.97; 95% CI = 2.06–7.64), dominant (p < 0.001; OR = 4.15; 95% CI = 2.85–6.06) and recessive (p = 0.00045; OR = 6.24; 95% CI = 2.24–17.36) (Fig. 2). Funnel plot showed asymmetry (Fig. S4), with an effect on the ES as indicated by the trim and fill analysis in recessive (estimated ES: OR = 3.74 [1.61–8.70], p = 0.002; number of trimmed studies: 2) genetic model (Table S7). However, the Egger’s and Begg’s test did not confirm the possible presence of a publication bias in the same model (Table S6). Moreover, even after correction for multiple comparisons, rs1800450 polymorphism showed strong association (p < 0.05) with RVVC risk in all the genetic models.

Moderator and Subgroup analysis: The effect of ethnicity on odds and heterogeneity for the pooled analysis of *MBL2* rs1800450 polymorphism and RVVC risk was studied (Table S8). Two case-control studies were found eligible for the analysis in each ethnic group including Asian, Caucasian and Mixed population, while only one study belongs to Egyptian population (Fig. S5). The ES did not change significantly based on the ethnicities of the included studies in any genetic model. The association of rs1800450 polymorphism with RVVC risk was re-evaluated in different ethnic groups (Table S8). The subgroup analysis identified the association rs1800450 polymorphism with increased risk of RVVC in Mixed ethnicity even after Bonferroni correction with no heterogeneity in all the genetic models including overall allelic contrast (p = 0.00001; OR = 3.46; 95% CI = 2.20–5.45), homozygous (p = 0.001; OR = 13.51; 95% CI = 3.03–60.22), heterozygous (p = 0.002; OR = 2.44; 95% CI = 1.40–4.26), dominant (p = 0.00001; OR = 3.23; 95% CI = 1.92–5.43) and recessive (p = 0.002; OR = 11.04; 95% CI = 2.49–48.88). Similarly, Caucasian group with 2 successful included case-control studies also showed an association of rs1800450 polymorphism with RVVC risk in overall allelic contrast (p = 0.002; OR = 6.49; 95% CI = 1.97–21.38), homozygous (p = 0.025; OR = 11.16; 95% CI = 1.35–92.10), heterozygous (p = 0.015; OR = 7.91; 95% CI = 1.51–41.53) and dominant (p < 0.001; OR = 7.14; 95% CI = 3.49–14.59) genetic models. However, this significance was lost (p > 0.05) for homozygous and heterozygous model after Bonferroni correction. Moreover, the heterogeneity was found in heterozygous and dominant genetic models for Caucasian group. In addition, no significant (p > 0.05) association of rs1800450 polymorphism with RVVC susceptibility was found in Asian ethnicity in any genetic models after correction for multiple testing (Table S8).

### Association between MBL2 rs1800450 polymorphism and RBV

**Figure 3:**
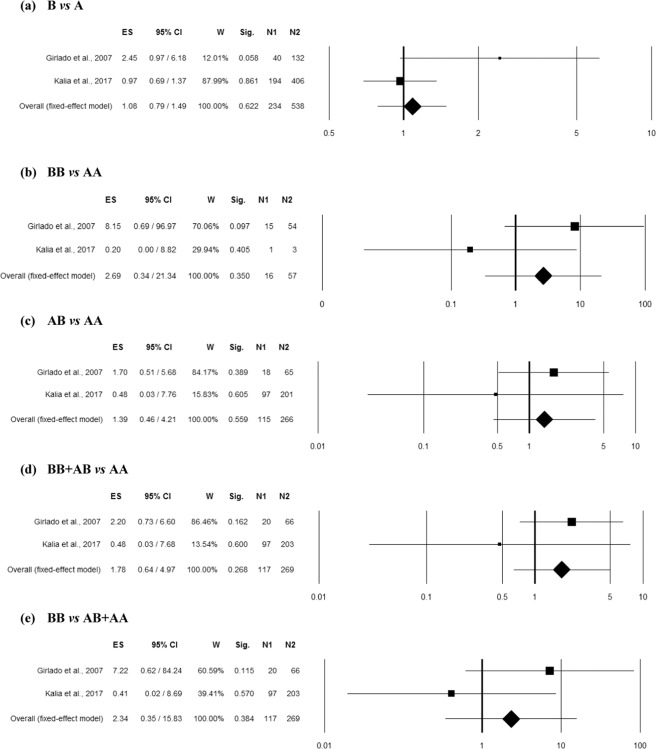
Forest plots for the association between rs1800450 (A/B = G/A allele) polymorphism and RBV under the (**a**) allelic, (**b**) homozygote, (**c**) heterozygote, (**d**) dominant and (**e**) recessive mode of inheritance for the studies including Girlado *et al*.^17^ and Kalia *et al*.^22^. Black boxes represents the value of odds ratio (OR) and the size of the boxes is inversely proportional to the size of the result study variance, so that more precise studies have larger boxes. Horizontal line is the 95% Confidence Interval (CI) of OR. The summary OR is represented by the diamond, where the center of the diamond indicates the OR. The ES is effect size expressed as OR; W, weight; Sig, statistical significance; N1, cases; N2, controls.

Two studies^17,22^ comprising 269 controls and 117 RBV cases belonging to two different ethnicities *i.e*. Asian and Mixed have reported an association of rs1800450 polymorphism with RBV and thus included in the combined analysis. No heterogeneity was observed among studies in overall allelic contrast (Q = 3.37, p = 0.066, I^2^ = 70.37%), homozygous (Q = 2.58, p = 0.108, I^2^ = 61.24%), heterozygous (Q = 0.67, p = 0.414, I^2^ = 0.00%), dominant (Q = 1.00, p = 0.316, I^2^ = 0.45%) and recessive (Q = 2.05, p = 0.152, I^2^ = 51.29%) genetic models. The overall ES indicated no significant association of rs1800450 polymorphism with RBV in any genetic models (Fig. 3).

### Association between MBL2 rs1800450 polymorphism and VVI

**Figure 4:**
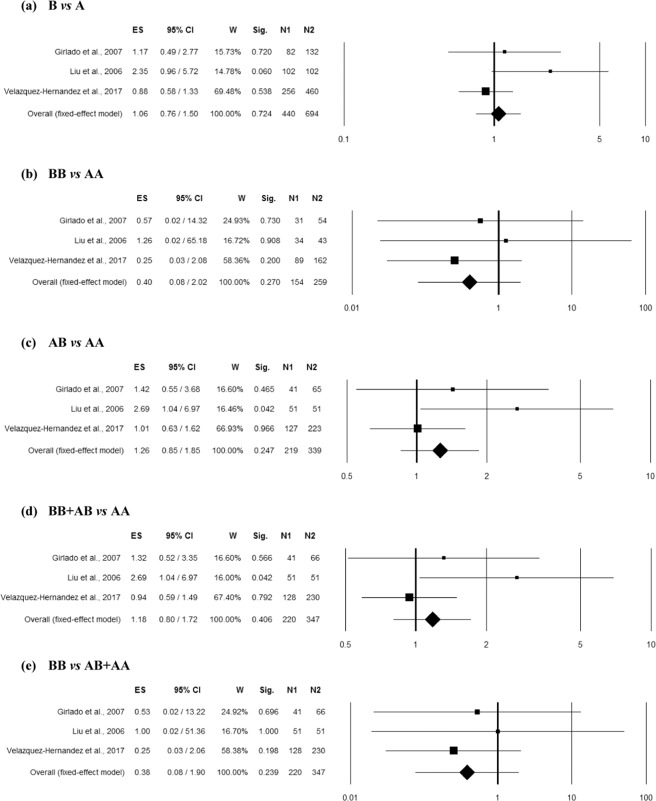
Forest plots for the association between rs1800450 (A/B = G/A allele) polymorphism and VVI under the (**a**) allelic, (**b**) homozygote, (**c**) heterozygote, (**d**) dominant and (**e**) recessive mode of inheritance for the studies including Girlado *et al*.^17^; Liu *et al*.^9^ and Velazquez-Hernandez *et al*.^20^. Black boxes represents the value of odds ratio (OR) and the size of the boxes is inversely proportional to the size of the result study variance, so that more precise studies have larger boxes. Horizontal line is the 95% Confidence Interval (CI) of OR. The summary OR is represented by the diamond, where the center of the diamond indicates the OR. The ES is effect size expressed as OR; W, weight; Sig, statistical significance; N1, cases; N2, controls.

A total of three reports^9,17,20^ provided the adequate data (including 347 controls and 220 VVI cases) to calculate the summary effect of rs1800450 polymorphism in susceptibility to VVI. No heterogeneity was observed among the included studies in any genetic models (Table S9). However, the overall ES indicated no significant association of *MBL2* rs1800450 polymorphism with VVI in any genetic models (Fig. 4). Funnel plot showed asymmetry in all the genetic models (Fig. S6). Therefore, the bias was reduced by trim and fill analysis leading to significant association of *MBL2* rs1800450 polymorphism with VVI in homozygous (estimated ES: OR = 0.25 [0.06–0.098], p = 0.046; number of trimmed studies: 2) and recessive (estimated ES: OR = 0.25 [0.06–0.097], p = 0.045; number of trimmed studies: 2) genetic models (Table S10). However, on Bonferroni correction, this emerged significance in both the models was lost. The Egger’s linear regression test confirms the possible presence of a publication bias in recessive genetic model (Table S9).

Moderator and Subgroup analysis: The effect of ethnicity on ES and heterogeneity between included studies was investigated (Table S11). Two case-control studies were found eligible for the analysis in mixed ethnic group, while only one study belongs to Asian ethnicity (Fig. S7). The ES did not change significantly based on the ethnicities of the included studies in any genetic models. The re-assessment based on mixed ethnicity depicted no possible association *MBL2* rs1800450 polymorphism with VVI, although the association was found significant for Asian group in heterozygous and dominant genetic models, which was lost after further analysis for multiple testing (Table S11).

### Association between MBL2 rs1800450 polymorphism and VVC

**Figure 5:**
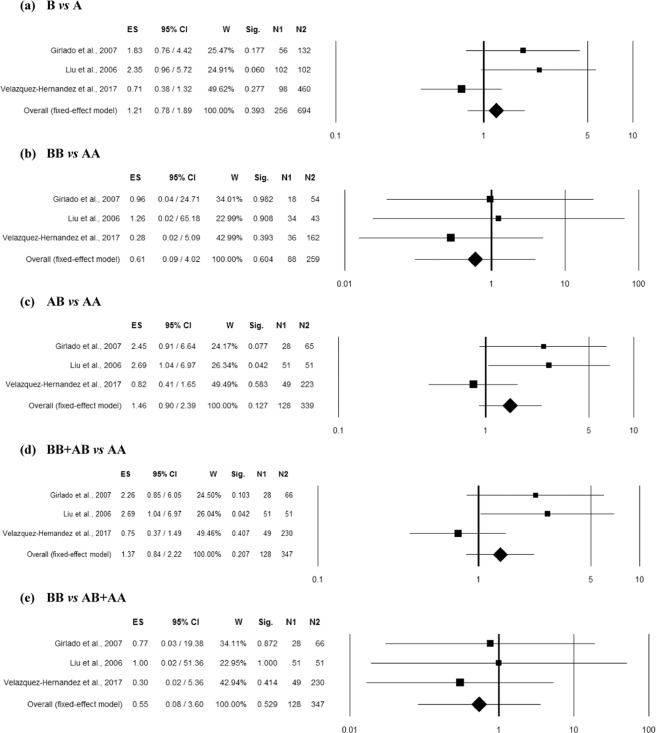
Forest plots for the association between rs1800450 (A/B = G/A allele) polymorphism and VVC under the (**a**) allelic, (**b**) homozygote, (**c**) heterozygote, (**d**) dominant and (**e**) recessive mode of inheritance for the studies includingGirlado *et al*.^17^; Liu *et al*.^9^ and Velazquez-Hernandez *et al*.^20^. Black boxes represents the value of odds ratio (OR) and the size of the boxes is inversely proportional to the size of the result study variance, so that more precise studieshave larger boxes. Horizontal line is the 95% Confidence Interval (CI) of OR. The summary OR is represented by the diamond, where the center of the diamond indicates the OR. The ES is effect size expressed as OR; W, weight; Sig, statistical significance; N1, cases; N2, controls.

Three case-control studies^9,17,20^ were pooled (including 347 controls and 128 VVC cases) for the analysis of association between *MBL2* rs1800450 polymorphism and VVC risk. No heterogeneity was found (Table S12). The overall ES indicated no significant association of *MBL2* rs1800450 polymorphism with VVC in any genetic model (Fig. 5). Funnel plot showed asymmetry (Fig. S8), with an effect on the ES as indicated by the trim and fill analysis in the allelic (estimated ES: OR = 0.71 [0.49–1.01], p = 0.059; number of trimmed studies: 2), heterozygous (estimated ES: OR = 0.82 [0.55–1.23], p = 0.339; number of trimmed studies: 2), Dominant (estimated ES: OR = 0.75 [0.50–1.11], p = 0.148; number of trimmed studies: 2) and recessive (estimated ES: OR = 0.30 [0.07–1.36], p = 0.118; number of trimmed studies: 2) genetic models (Table S13). The Egger’s and Begg-Mazumdar’s tests did not confirm the presence of a publication bias in any genetic model (Table S12).

Moderator and Subgroup analysis: The effect of ethnicity on the pooled results was investigated, wherein two case-control studies were found eligible for the analysis in mixed ethnic group, while only one study belongs to Asian ethnicity (Fig. S9). The ES did not change significantly based on the ethnicities of the included studies in any genetic model (Table S14). We re-accessed the possible link of *MBL2* rs1800450 polymorphism with VVC in Mixed and Asian ethnic groups, however the association was observed for Asian ethnicity only in heterozygous and dominant genetic models, which was lost after further analysis for multiple testing (Table S14).

### Association between MBL2 rs1800450 polymorphism and BV

**Figure 6:**
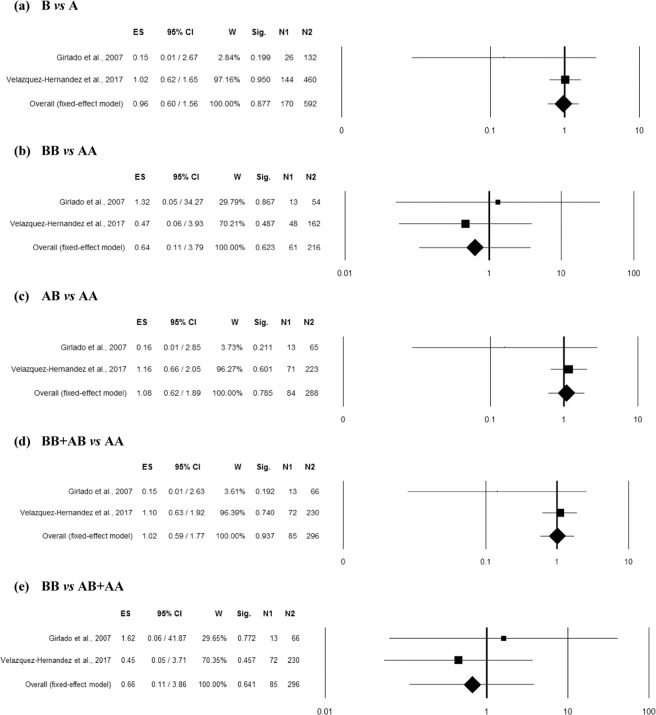
Forest plots for the association between rs1800450 (A/B = G/A allele) polymorphism and BV under the (**a**) allelic, (**b**) homozygote, (**c**) heterozygote, (**d**) dominant and (**e**) recessive mode of inheritance for the studies including Girlado *et al*.^17^ and Velazquez-Hernandez *et al*.^20^. Black boxes represents the value of odds ratio (OR) and the size of the boxes is inversely proportional to the size of the result study variance, so that more precise studieshave larger boxes. Horizontal line is the 95% Confidence Interval (CI) of OR. The summary OR is represented by the diamond, where the center of the diamond indicates the OR. The ES is effect size expressed as OR; W, weight; Sig, statistical significance; N1, cases; N2, controls.

Two studies^17,20^ including 292 controls and 85 BV cases of mixed ethnicity reported an association of rs1800450 polymorphism with BV and thus included in the combined analysis. No heterogeneity was observed among studies in overall allelic contrast (Q = 1.63, p = 0.201, I^2^ = 38.74%), homozygous (Q = 0.27, p = 0.603, I^2^ = 0.00%), heterozygous (Q = 1.76, p = 0.184, I^2^ = 43.22%), dominant (Q = 1.80, p = 0.179, I^2^ = 44.55%) and recessive (Q = 0.42, p = 0.517, I^2^ = 0.00%) genetic models. The overall ES did not show significant association of rs1800450 polymorphism with BV in any genetic model (Fig. 6).

### Association between MBL2 combined Exon1 polymorphisms (rs5030737, rs1800450, rs1800451) and RVVI

**Figure 7:**
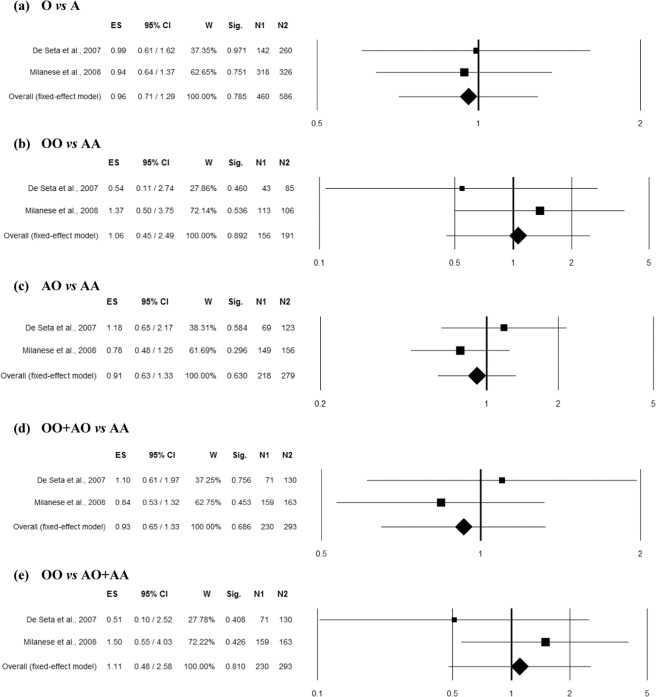
Forest plots for the association between combined Exon1 polymorphisms (involving A/O allele = C,G,G/T,A,A alleles of rs5030737, rs1800450 and rs1800451 polymorphisms respectively) and RVVI under the (**a**) allelic, (**b**) homozygote, (**c**) heterozygote, (**d**) dominant and (**e**) recessive mode of inheritance for the studies including De Seta *et al*.^16^ and Milanese *et al*.^10^. Black boxes represents the value of odds ratio (OR) and the size of the boxes is inversely proportional to the size of the result study variance, so that more precise studieshave larger boxes. Horizontal line is the 95% Confidence Interval (CI) of OR. The summary OR is represented by the diamond, where the center of the diamond indicates the OR. The ES is effect size expressed as OR; W, weight; Sig, statistical significance; N1, cases; N2, controls.

Two studies^10,16^ comprising 293 controls and 230 RVVI cases of Caucasian ethnicity reported an association of rs1800450 polymorphism with RVVI and thus included in the combined analysis. No heterogeneity was observed among studies in overall allelic contrast (Q = 0.03, p = 0.868, I^2^ = 0.00%), homozygous (Q = 0.91, p = 0.340, I^2^ = 0.00%), heterozygous (Q = 1.16, p = 0.282, I^2^ = 13.67%), dominant (Q = 0.50, p = 0.482, I^2^ = 0.00%) and recessive (Q = 1.26, p = 0.262, I^2^ = 20.60%) genetic models. The overall ES did not show significant association of rs1800450 polymorphism with RVVI in any genetic model (Fig. 7).

### Combined Exon1 polymorphisms and RBV

**Figure 8:**
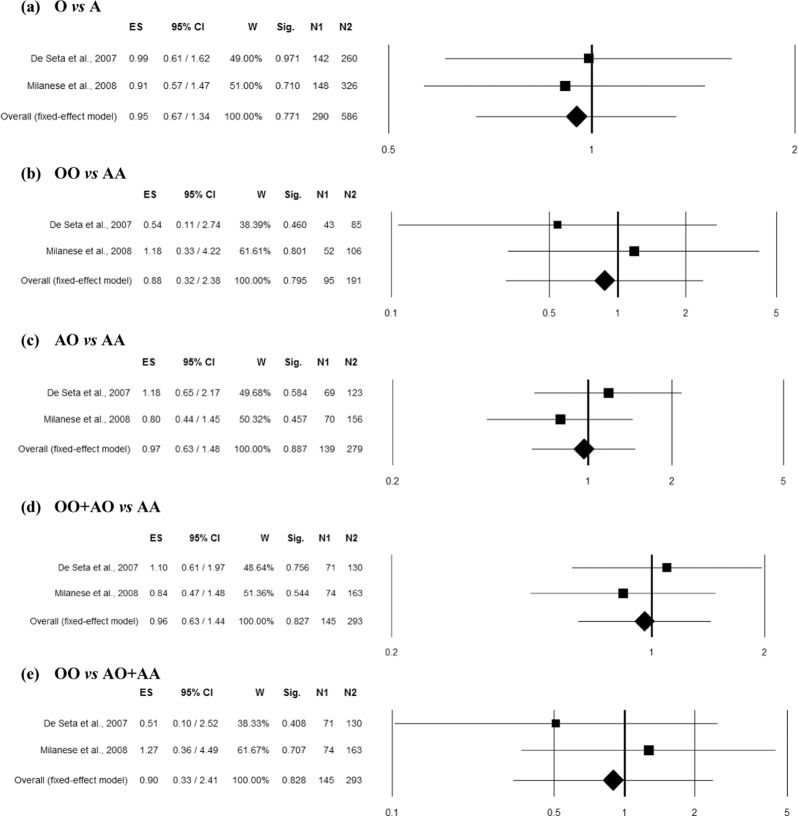
Forest plots for the association between combined Exon1 polymorphisms (involving A/O allele = C,G,G/T,A,A alleles of rs5030737, rs1800450 and rs1800451 polymorphisms respectively) and RBV under the (**a**) allelic, (**b**) homozygote, (**c**) heterozygote, (**d**) dominant and (**e**) recessive mode of inheritance for the studies including De Seta *et al*.^16^ and Milanese *et al*.^10^. Black boxes represents the value of odds ratio (OR) and the size of the boxes is inversely proportional to the size of the result study variance, so that more precise studieshave larger boxes. Horizontal line is the 95% Confidence Interval (CI) of OR. The summary OR is represented by the diamond, where the center of the diamond indicates the OR. The ES is effect size expressed as OR; W, weight; Sig, statistical significance; N1, cases; N2, controls.

Two studies^10,16^ comprising 293 controls and 145 RBV cases of Caucasian ethnicity reported an association of rs1800450 polymorphism with RVVI and thus included in the combined analysis. No heterogeneity was observed among studies in overall allelic contrast (Q = 0.05, p = 0.815, I^2^ = 0.00%), homozygous (Q = 0.54, p = 0.461, I^2^ = 0.00%), heterozygous (Q = 0.83, p = 0.362, I^2^ = 0.00%), dominant (Q = 0.42, p = 0.518, I^2^ = 0.00%) and recessive (Q = 0.78, p = 0.378, I^2^ = 0.00%) genetic models. The overall ES did not show significant association of rs1800450 polymorphism with RVVI in any genetic model (Fig. 8).

### Sensitivity analysis

In order to examine the overall strength of pooled assessments, a leave-one-out sensitivity analysis was performed by eliminating one study at a time and re-computing the summarized ES of each association. The analysis showed no major variation between the re-computed and original values, warranting the stability of meta-analysed results (Fig. S10-S13, Fig. S15-S17). However, statistical significance for the association of rs1800450 polymorphism with VVC originates in allelic (OR = 2.07, 95% CI = 1.11–3.87, p = 0.022), heterozygous (OR = 2.57, 95% CI = 1.29–5.12, p = 0.007) and dominant (OR = 2.47, 95% CI = 1.25–4.90, p = 0.009) genetic models, when the analysis was performed by removing study by Velazquez-Hernandez *et al*.^20^. (Fig. S14).

### MBL2 rs1800451, rs11003125, rs7096206, rs7095891, rs10824792, rs2120132, rs2120131, rs2165813, rs2099903, rs2099902, rs11003124, rs7084554, rs36014597 and rs11003123 polymorphisms

There were two studies^17,20^ that have evaluated the effect of rs1800451 polymorphism, however, the outcome of interest in both studies were different, which limited the further meta-analysis. Similarly, *MBL2* promoter SNPs including rs11003125, rs7096206, rs7095891 were evaluated by single study *i.e*. Kalia *et al*.^22^, that has suggested the risk effect of rs7096206 polymorphism in association with RVVI. Moreover, 3′UTR variants including rs10824792, rs2120132, rs2120131, rs2165813, rs2099903 and rs2099902 of *MBL2* were evaluated by single study *i.e*. Kalia *et al*.^23^, where in only rs10824792 polymorphism was found to be increasing the odds of RVVI susceptibility. In addition, other *MBL2* promoter variants including rs11003124, rs7084554, rs36014597 and rs11003123 were evaluated by one study *i.e*. Kalia *et al*.^24^, in which rs7084554 and rs36014597 SNPs were found to be predisposing women to RVVI. Therefore, scarcity of literary evidences for these polymorphisms limited the evaluation of their association with RVVI by quantitative synthesis.

### Availability of data and materials

The data that support the findings of this study are available from the corresponding author upon reasonable request.

## Discussion

This is the first comprehensive and systematic meta-analysis undertaken for the evaluation of *MBL2* gene polymorphisms in susceptibility to acute and recurrent nature of vaginal infections. Total 12 reports^8–10,16–24^ with clear descriptions of study characteristics were included in the present study. These studies were further subjected to different quantitative synthesis considering the cases of RVVI and/or VVI and the results have been summarized in Table 2. An ethnicity based sub-group analyses was also performed for the better assessment of the results. Total five different genetic models including allelic, homozygote, heterozygote, dominant and recessive were analyzed for each comparison. On the basis of seven independent publications^8,9,17–19,21,22^, our meta-analysis clearly produced statistical evidence that rs1800450 polymorphism is significantly associated with an increased risk of RVVI as indicated by all the studied genetic models. The essential attribute of the genetic variations is that their incidence may show a divergence among different ethnic groups, as for instance in the present study, the rs1800450 polymorphism was found to be responsible for increasing the odds of RVVI risk in the Caucasian as well as Mixed ethnicity, while no such significant association was found in the Asian group. Moreover, the data analysis showed the presence of significant heterogeneity between studies in different genetic models. Therefore, an advance attempt was made to found whether the ethnicity is responsible factor for the observed heterogeneity or not. However, moderator analysis rejected the assumption suggesting other factors *e.g*. demographic or milieu based, that might be responsible for the observed heterogeneity between studies.

**Table 2:** Summarised meta-analysis results of *MBL2* polymorphisms and (R)VVI risk.

SNP and (R)VVI	Comparisons	Heterogeneity analysis			Effect Model	Egger’s test (*p*_*e*_)	Begg’s test (*p*_*b*_)	Overall effect size (Trimmed)			*p*_*c*_
		Q-value	P_heterogeneity_	I^2^ (%)				OR	CI	p-value	
rs1800450 and RVVI	B *vs* A	50.10	<0.0001	88.02	R	0.002	0.293	**3.54**	1.69–7.43	0.001	0.005
	BB *vs* AA	4.96	0.549	0.00	F	0.380	0.176	**8.12**	2.94–22.4	5.3 × 10^−5^	2.6 × 10^−4^
	AB *vs* AA	14.11	0.028	57.49	R	0.348	0.652	**3.92**	2.05–7.50	4.0 × 10^−5^	2.0 × 10^−4^
	BB + AB *vs* AA	12.74	0.047	52.91	R	0.406	0.652	**4.54**	2.50–8.24	6.9 × 10^−7^	3.2 × 10^−6^
	BB *vs* AB + AA	6.24	0.397	3.85	F	0.367	0.176	2.76	1.19–6.37	0.018	0.09
rs1800450 and RVVC	B *vs* A	34.93	<0.0001	82.82	R	0.012	0.176	**3.54**	1.79–7.00	2.0 × 10^−4^	0.001
	BB *vs* AA	0.79	0.977	0.00	F	0.910	0.851	**12.22**	4.09–36.5	7.4 × 10^−6^	3.7 × 10^−5^
	AB *vs* AA	13.77	0.032	56.44	R	0.441	0.652	**3.97**	2.06–7.64	3.7 × 10^−5^	1.8 × 10^−4^
	BB + AB *vs* AA	12.44	0.053	51.77	F	0.493	0.652	**4.15**	2.85–6.06	1.5 × 10^−13^	7.5 × 10^−13^
	BB *vs* AB + AA	2.85	0.827	0.00	F	0.270	0.176	**3.74**	1.61–8.70	0.002	0.01
rs1800450 and RBV	B *vs* A	3.37	0.066	70.37	F	–	–	1.08	0.79–1.49	0.622	3.11
	BB *vs* AA	2.58	0.108	61.24	F	–	–	2.69	0.34–21.34	0.350	1.75
	AB *vs* AA	0.67	0.414	0.00	F	–	–	1.39	0.46–4.21	0.559	2.79
	BB + AB *vs* AA	1.00	0.316	0.45	F	–	–	1.78	0.64–4.97	0.268	1.34
	BB *vs* AB + AA	2.05	0.152	51.29	F	–	–	2.34	0.35–15.83	0.384	1.92
rs1800450 and VVI	B *vs* A	3.92	0.141	49.01	F	0.353	0.117	0.88	0.65–1.19	0.399	1.995
	BB *vs* AA	0.55	0.758	0.00	F	0.070	0.117	0.25	0.06–0.98	0.046	0.23
	AB *vs* AA	3.33	0.189	39.94	F	0.335	0.117	1.01	0.72–1.41	0.953	4.765
	BB + AB *vs* AA	3.84	0.146	47.94	F	0.345	0.117	0.94	0.67–1.31	0.711	3.555
	BB *vs* AB + AA	0.42	0.810	0.00	F	0.048	0.117	0.25	0.06–0.97	0.045	0.225
rs1800450 and VVC	B *vs* A	5.81	0.055	65.57	F	0.086	0.117	0.71	0.49–1.01	0.059	0.295
	BB *vs* AA	0.48	0.788	0.00	F	0.341	0.117	0.61	0.09–4.02	0.604	3.02
	AB *vs* AA	5.23	0.073	61.75	F	0.104	0.602	0.82	0.55–1.23	0.339	1.695
	BB + AB *vs* AA	5.89	0.053	66.04	F	0.113	0.602	0.75	0.50–1.11	0.148	0.74
	BB *vs* AB + AA	0.30	0.862	0.00	F	0.322	0.117	0.30	0.07–1.36	0.118	0.59
rs1800450 and BV	B *vs* A	1.63	0.201	38.74	F	–	–	0.96	0.60–1.56	0.877	4.385
	BB *vs* AA	0.27	0.603	0.00	F	–	–	0.64	0.11–3.79	0.623	3.115
	AB *vs* AA	1.76	0.184	43.22	F	–	–	1.08	0.62–1.89	0.785	3.925
	BB + AB *vs* AA	1.80	0.179	44.55	F	–	–	1.02	0.59–1.77	0.937	4.685
	BB *vs* AB + AA	0.42	0.517	0.00	F	–	–	0.66	0.11–3.86	0.641	3.205
Combined Exon1 and RVVI	O *vs* A	0.03	0.868	0.00	F	–	–	0.96	0.71–1.29	0.785	0.392
	OO *vs* AA	0.91	0.340	0.00	F	–	–	1.06	0.45–2.49	0.892	4.46
	AO *vs* AA	1.16	0.282	13.67	F	–	–	0.91	0.63–1.33	0.630	3.15
	OO + AO *vs* AA	0.50	0.482	0.00	F	–	–	0.93	0.65–1.33	0.686	3.43
	OO *vs* AO + AA	1.26	0.262	20.60	F	–	–	1.11	0.48–2.58	0.810	4.05
Combined Exon1 and RBV	O *vs* A	0.05	0.815	0.00	F	–	–	0.95	0.67–1.34	0.771	3.855
	OO *vs* AA	0.54	0.461	0.00	F	–	–	0.88	0.32–2.38	0.795	3.975
	AO *vs* AA	0.83	0.362	0.00	F	–	–	0.97	0.63–1.48	0.887	4.435
	OO + AO *vs* AA	0.42	0.518	0.00	F	–	–	0.96	0.63–1.44	0.827	4.135
	OO *vs* AO + AA	0.78	0.378	0.00	F	–	–	0.90	0.33–2.41	0.828	4.14

rs1800450 polymorphism (involving A/B allele = G/A allele), combined Exon1 polymorphisms (involving A/O allele = C,G,G/T,A,A alleles of rs5030737, rs1800450 and rs1800451 polymorphisms respectively),R = Random effect model, F = Fixed effect model, *p*_*e*_ = Egger’s test p-value, *p*_*b*_ = Begg’s test p-value, OR = Odds ratio, CI = 95% confidence intervals, *p*_c_ = p-value after Bonferroni correction *i.e*. p-value × 5 genetic models, Bold numbers indicates statistically significant OR values after Bonferroni correction.

On quantitative synthesis based on RVVI categories *i.e*. RVVC and RBV, the heterogeneity reduced to two genetic models for RVVC, while none for RBV, the former included the same seven independent publications as that of RVVI but with different sample size while the latter included two independent studies^17,22^ belonging to different ethnic groups. However, the pooled analysis showed rs1800450 polymorphism was associated with risk of RVVC even after correction for multiple testing, while no such association was found with RBV even though the sample size was sufficient enough to explore the relationship. Furthermore, the rs1800450 and RVVC association was found to be significant in the ethnicities involving Caucasian and Mixed. These results are in line with the findings of earlier meta-analysis^32^, conducted six years ago that has 5 studies in common with present analysis stating that *MBL2* codon 54 gene polymorphism is significantly associated with recurrent vaginal fungal infections.

On the other hand, meta-analysis of three independent publications^9,17,20^, showed no significant association of *MBL2* rs1800450 polymorphism with VVI in any genetic model, though stratification based on ethnicity revealed an increased odds of getting VVI in Asian ethnicity than the mixed racial group. However, careful consideration should be taken regarding the interpretation of these results as there was only one study for Asian ethnicity and the significance was lost after further analysis for multiple testing. Moreover, on reduction of bias by Trim-fill analysis^31^, a significant association of *MBL2* rs1800450 polymorphism in providing protection against VVI originated in homozygous and recessive mode of inheritance. In consonance to these findings, a study suggested an active involvement of MBL in providing protection against VVC but not against RVVC in women carrying *MBL2* variant genotype^9^. The difference may possibly be due to variant allele oligomers, which are recommended to be functionally inactive and degradable comparative to wild allele oligomers^33^. Therefore, MBL concentration increases during the first attack of *Candida* vaginitis, however with recurrent attacks the MBL did not remain sturdy enough to provide protection against the disease owing to variant monomers synthesis. However, careful consideration should be taken regarding the interpretation of these results as the emerged significance was lost after correction for multiple testing.

In absence of inter-study heterogeneity, stratified meta-analysis based on VVC and BV revealed no significant association with codon 54 polymorphism, the former includes the same three independent publications as that of VVI but with different sample size while the later includes two independent studies^17,20^ belonging to same ethnicity *i.e*. Mixed. However, sensitivity analysis indicated the lack of robustness of pooled estimates for codon 54 and VVC association, because significant association under allelic, heterozygous and dominant model of inheritance for VVC risk originated when the study by Velazquez-Hernandez *et al*.^20^ was omitted from the meta-analysis. This is in agreement with the result of previous meta-analysis^32^, conducted six years ago involving all the studies in common except one excluded by sensitivity analysis, stating that *MBL2* codon 54 gene polymorphism is considerably linked with the risk of acute vaginal fungal infections. However, in this regard, it is worth mentioning that the present quantitative synthesis is an updated analysis with sufficient power (474 participants) due to the inclusion of newly published literature than the previous meta-analysis (175 participants) to precisely access the role of rs1800450 polymorphism in (R)VVC. Nevertheless, the study^20^, used Taqman detection method, which has been suggested of producing false positive results, therefore the results should be interpreted with care^34^.

Meta-analysis of combined Exon1 polymorphisms with RVVI (total 523 participants) and BV (total 438 participants) involving two independent studies^10,16^ of same ethnicity (Caucasian) as well as genotyping method (Melting temperature assay) did not show any notable association either with enhanced or reduced risk of disease. Though, the sample size was large enough, the result for this analysis might have experienced low power accredited to the low Global MAF (0.02, as per the 1000 genome project) of *MBL2* rs5030737 of Exon 1 variant. In addition, other MBL2 genetic polymorphisms, rs1800451, rs11003125, rs7096206, rs7095891, rs10824792, rs2120132, rs2120131, rs2165813, rs2099903, rs2099902, rs11003124, rs7084554, rs36014597 and rs11003123 could not be investigated due to the absence of multiple studies in response to the same infection. Nevertheless, studying the combined effect of *MBL2* structural and promoter gene variations in pathogenesis of (R)VVI is important, which further assures the study to be performed in a generously proportioned scale given that more studies regarding the association should be available in the literature.

Although, meticulous methodology was used for the systematic evaluation of results, yet the present analysis has some limitations to be cared for. Firstly, the study might have experienced the publication bias owing to the inclusion of English literature only, which could have limited the published evidences. Secondly, sensitivity analysis indicated that the statistical significance of rs1800450 in association with VVC altered on exclusion of a study, further suggesting that the pooled estimates for this association may be short on strength. Third, the control group of study by Kalia *et al*.^22^ was not in accordance with HWE, which may be attributed to the population stratification/selection pressure or the reason could be other prevailing infectious diseases or genotyping error. However, it is of note that, omission of this study^22^, did not alter the results of quantitative synthesis, suggesting the robustness of results. Fourth, our pooled ORs were based on un-adjusted data for potential confounding factors, such as the well-known risk factors for mucosal vaginal infection, which might have affected the accuracy of the data, though no sufficient information was available in this regards. Fifth, some ethnicity based sub-group analyses, might lack sufficient statistical power to identify the authentic relationship in different ethnic groups and hence demands more such studies to be published for the up-dated quantitative synthesis. Finally, the caveat for dealing with multiple comparisons with Bonferroni correction is the risk for false negative outcomes hence, caution should be taken while interpreting the results.

Albeit of aforementioned limitations, this meta-analysis offered several substantial advantages to the field. Firstly, the included studies were stringently checked for quality assessment by applying NOS scale and each study found to be of good quality with clear descriptions of the inclusion criteria. Secondly, in order to achieve consistent results, a meticulous procedure for the identification of study, statistical methodology adopted, and data selection was used to minimize the possibility of bias. Moreover, the analysis of publication bias was performed using the three well known criteria and the two of them indicated no bias in the majority of results. Third, almost all the studies involved in the present meta-analysis used one mode of genotyping *i.e*. PCR-RFLP, which is important to avoid variation in results that may arise due to differences in performance and sensitivity of different detection methods. Fourth, except for one comparison, the sensitivity analysis showed potency in the present study’s conclusions. Fifth, the random-effect model was used to minimize the problem that may arise due to the observed inconsistency by obtaining wider confidence intervals. Hence, the present study may contribute significantly to the understanding of (R)VVI pathogenesis.

## Conclusions

In conclusion, the present investigation evaluated 12 case-control association studies, whose quantitative synthesis provided strong evidence in favour of rs1800450 polymorphism as a genetic predisposing factor for RVVI and RVVC in various ethnic groups. Interestingly, no evidence of association was found between the combined effect of Exon 1 polymorphisms and (R)VVI. Moreover, the lack of sufficient literary evidences for many of the important polymorphisms of *MBL2* including 5′-promoter and 3′-UTR variants in response to (R)VVI, restricted the pooled analysis of these variants. Thus, the study demands more such studies to confirm MBL substitution as novel immunotherapeutic approach in order to replace traditional non-specific anti(R)VVI treatments. Furthermore, future studies involving the extensive screening of *MBL2* variants with regard to (R)VVI susceptibility are warranted to decode the potential molecular markers underlying the pathogenesis.

## Supplementary information

Supplementary information.
