# Supplementary material for: A meta-analysis of mannose-binding lectin gene polymorphisms with the risk of recurrent vulvovaginal infections
Source: Sci Rep. 2020 Apr 8;10:6079. doi: 10.1038/s41598-020-63261-8 (PMC7142065; doi:10.1038/s41598-020-63261-8)
Supplement: Supplementary file 1 — Supplementary information. [file 41598_2020_63261_MOESM1_ESM.pdf]

A meta-analysis of mannose-binding lectin gene polymorphisms with the risk of recurrent vulvovaginal infections

Namarta Kalia[a]*,Jatinder Singh[a], Akash Kumar Rauniyar[b], Manpreet Kaur[c]*

[a]Department of Molecular Biology & Biochemistry, Guru Nanak Dev University, Amritsar, India.

[b]Department of Information Technology, University of Turku, Turku, Finland.

[c]Department of Human Genetics, Guru Nanak Dev University, Amritsar, India.

* Corresponding authors

- Namarta Kalia; Email: kalianamarta62@gmail.com
- Manpreet Kaur; Email: dr.manpreetdhuna@gmail.com

Total words excluding abstract and references = 5032

SUPPLEMENTARY FIGURES

FIG. S1

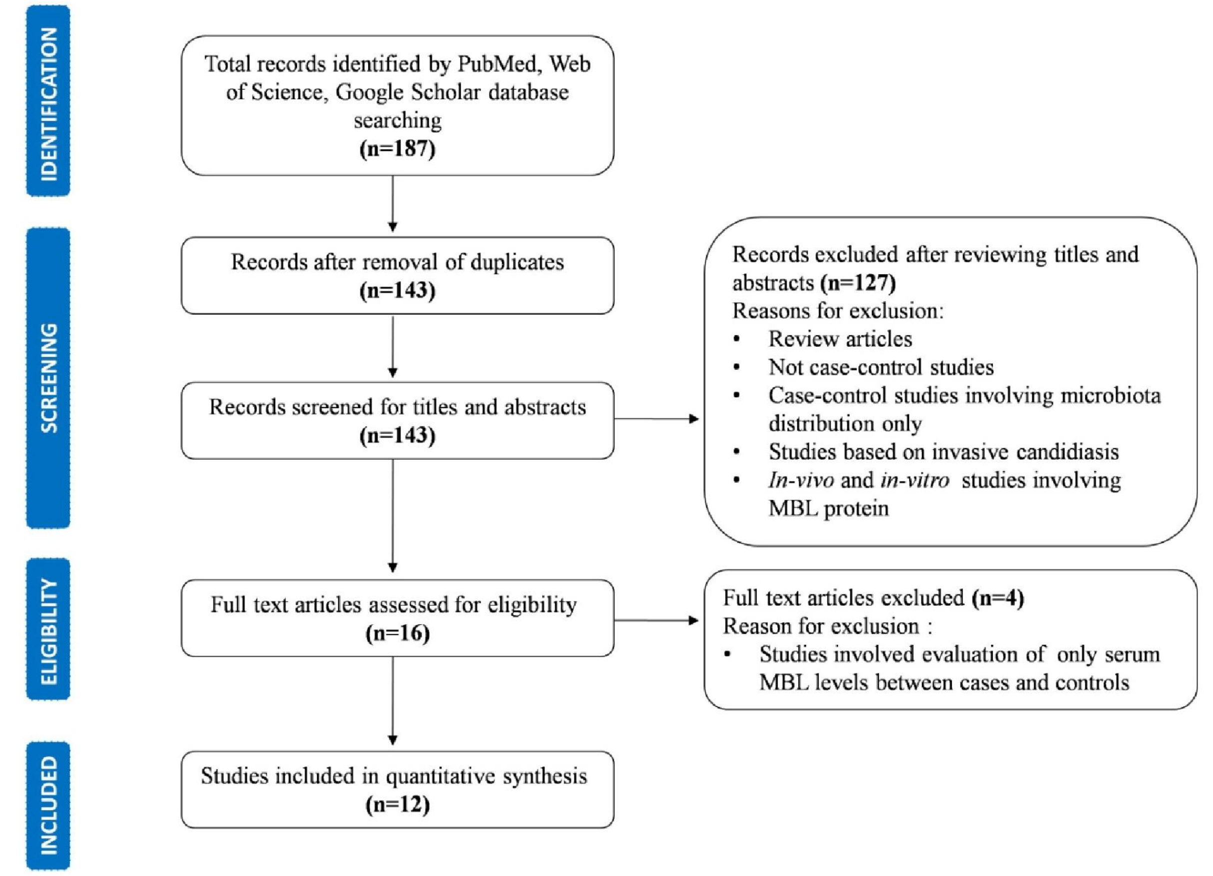

FIG S1 | Preferred Reporting Items for Systematic Reviews and Meta-Analysis (PRISMA) flow chart for literature search and study selection in the present meta-analysis.

FIG. S2

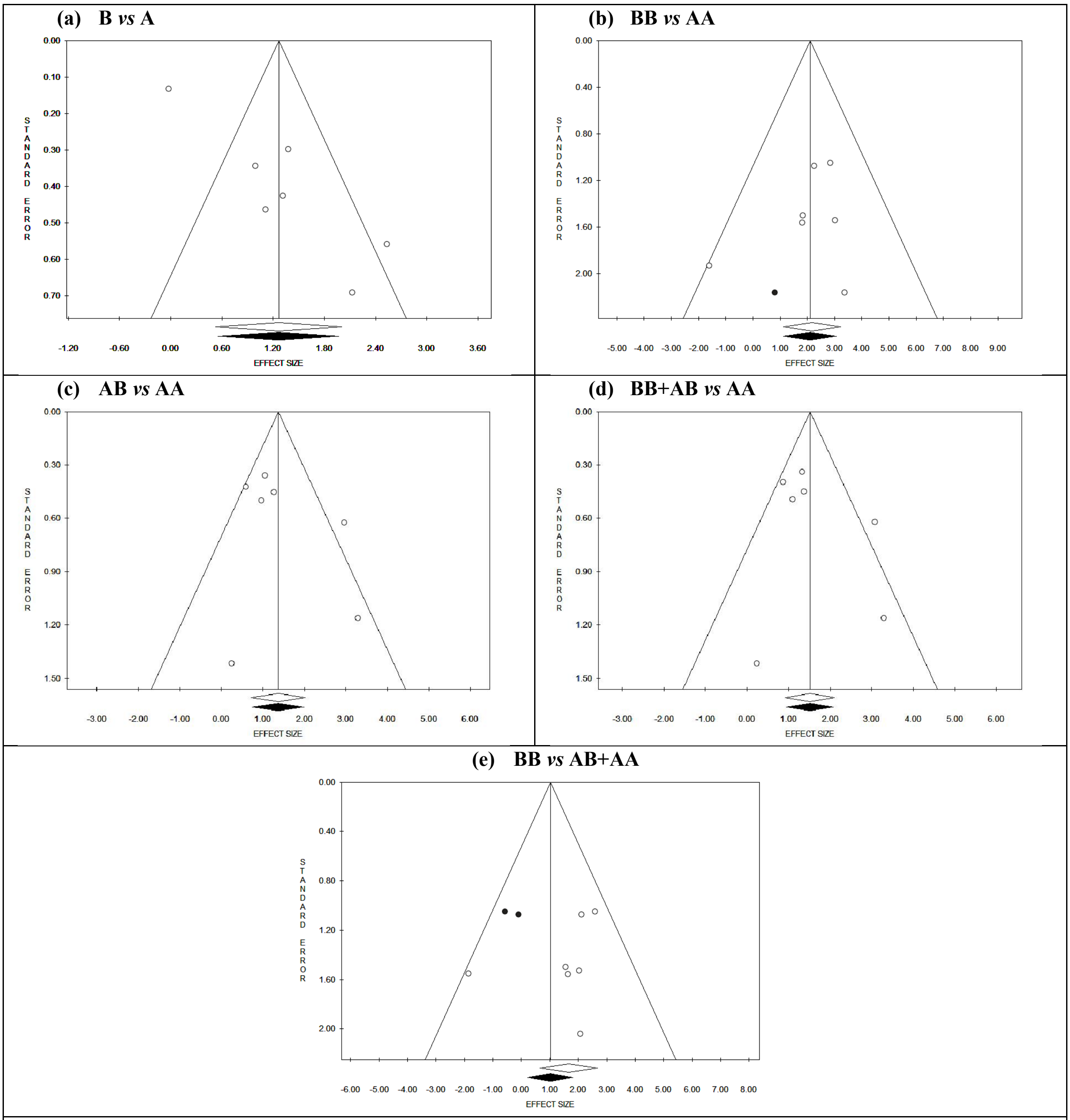

FIG. S2 | **Funnel plot and trim-and-fill analysis of studies that evaluated the** association between rs1800450 (A/B=G/A allele) polymorphism and RVVI. Effect Size (OR) against standard error in (a) Allelic, (b) homozygote, (c) heterozygote, (d) dominant and (e) recessive genetic models.

FIG. S3

(a) B *vs* A

	ES	95% CI	W	Sig.
Kalia et al., 2017	0.98	0.76 / 1.27	55.01%	0.894
Liu et al., 2006	8.39	2.16 / 32.56	44.99%	0.002
Asian	2.58	0.32 / 20.88	100.00%	0.375
Babula et al., 2003	12.62	4.22 / 37.75	45.58%	0.000
Donders et al., 2008	3.72	1.62 / 8.57	54.42%	0.002
Caucasian	6.49	1.97 / 21.38	100.00%	0.002
Hammad et al., 2018	3.04	1.23 / 7.55	100.00%	0.016
Egyptian	3.04	1.23 / 7.55	100.00%	0.016
Girlado et al., 2007	2.70	1.38 / 5.31	43.02%	0.004
Wojitani et al., 2012	3.98	2.21 / 7.16	56.98%	0.000
Mixed	3.37	2.16 / 5.25	100.00%	0.000

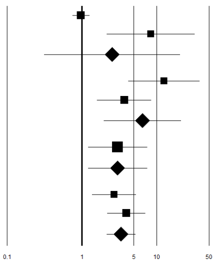

(b) BB *vs* AA

	ES	95% CI	W	Sig.
Kalia et al., 2017	0.20	0.00 / 8.82	51.93%	0.405
Liu et al., 2006	29.00	0.42 / 2021.75	48.07%	0.120
Asian	2.19	0.02 / 286.09	100.00%	0.753
Babula et al., 2003	20.48	0.99 / 422.61	48.62%	0.051
Donders et al., 2008	6.28	0.33 / 119.36	51.38%	0.221
Caucasian	11.16	1.35 / 92.10	100.00%	0.025
Hammad et al., 2018	6.18	0.29 / 132.15	100.00%	0.244
Egyptian	6.18	0.29 / 132.15	100.00%	0.244
Girlado et al., 2007	9.64	1.16 / 80.04	48.81%	0.036
Wojitani et al., 2012	17.38	2.20 / 137.35	51.19%	0.007
Mixed	13.03	2.97 / 57.19	100.00%	0.001

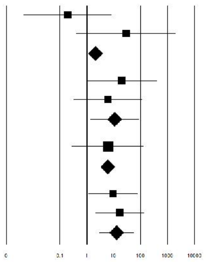

(c) AB *vs* AA

	ES	95% CI	W	Sig.
Kalia et al., 2017	1.28	0.08 / 20.67	46.43%	0.860
Liu et al., 2006	26.88	2.76 / 261.65	53.57%	0.005
Asian	6.55	0.34 / 127.94	100.00%	0.215
Babula et al., 2003	19.50	5.72 / 66.42	46.78%	0.000
Donders et al., 2008	3.58	1.47 / 8.71	53.22%	0.005
Caucasian	7.91	1.51 / 41.53	100.00%	0.015
Hammad et al., 2018	2.65	0.99 / 7.10	100.00%	0.052
Egyptian	2.65	0.99 / 7.10	100.00%	0.052
Girlado et al., 2007	1.81	0.79 / 4.15	41.71%	0.164
Wojitani et al., 2012	2.90	1.43 / 5.86	58.29%	0.003
Mixed	2.38	1.39 / 4.07	100.00%	0.002

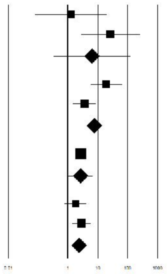

(d) BB+AB *vs* AA

	ES	95% CI	W	Sig.
Kalia et al., 2017	1.27	0.08 / 20.47	46.45%	0.865
Liu et al., 2006	26.88	2.76 / 261.65	53.55%	0.005
Asian	6.52	0.33 / 128.52	100.00%	0.218
Babula et al., 2003	21.75	6.43 / 73.62	46.82%	0.000
Donders et al., 2008	3.98	1.64 / 9.62	53.18%	0.002
Caucasian	8.81	1.67 / 46.43	100.00%	0.010
Hammad et al., 2018	3.01	1.14 / 7.93	100.00%	0.026
Egyptian	3.01	1.14 / 7.93	100.00%	0.026
Girlado et al., 2007	2.41	1.11 / 5.24	42.38%	0.026
Wojitani et al., 2012	3.80	1.95 / 7.40	57.62%	0.000
Mixed	3.13	1.89 / 5.19	100.00%	0.000

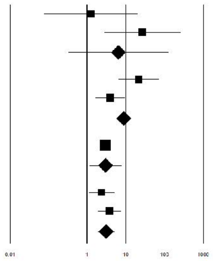

(e) BB *vs* AB+AA

	ES	95% CI	W	Sig.
Kalia et al., 2017	0.16	0.01 / 3.27	63.40%	0.231
Liu et al., 2006	7.92	0.14 / 434.29	36.60%	0.311
Asian	0.66	0.06 / 7.40	100.00%	0.733
Babula et al., 2003	7.71	0.39 / 153.96	49.08%	0.181
Donders et al., 2008	4.73	0.25 / 89.54	50.92%	0.300
Caucasian	6.01	0.74 / 49.00	100.00%	0.094
Hammad et al., 2018	5.17	0.24 / 110.12	100.00%	0.292
Egyptian	5.17	0.24 / 110.12	100.00%	0.292
Girlado et al., 2007	8.39	1.02 / 69.02	48.86%	0.048
Wojitani et al., 2012	13.50	1.72 / 105.93	51.14%	0.013
Mixed	10.70	2.45 / 46.68	100.00%	0.002

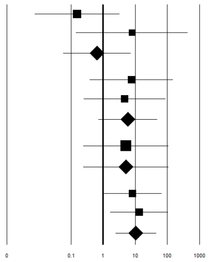

FIG. S3 | Subgroup analysis performed on the basis of ethnicity for the association between rs1800450 (A/B=G/A allele) polymorphism and RVVI under the (a) allelic, (b) homozygote, (c) heterozygote, (d) dominant and (e) recessive mode of inheritance for the studies including Babula *et al*., 2003 [8]; Donders *et al*., 2008 [18]; Girlado *et al*., 2007 [17]; Hammad *et al*., 2018 [21]; Kalia *et al*., 2017 [22]; Liu *et al*., 2006 [9] and Wojitani *et al*., 2012 [19]. ES, effect size (OR); Sig, statistical significance.

FIG. S4

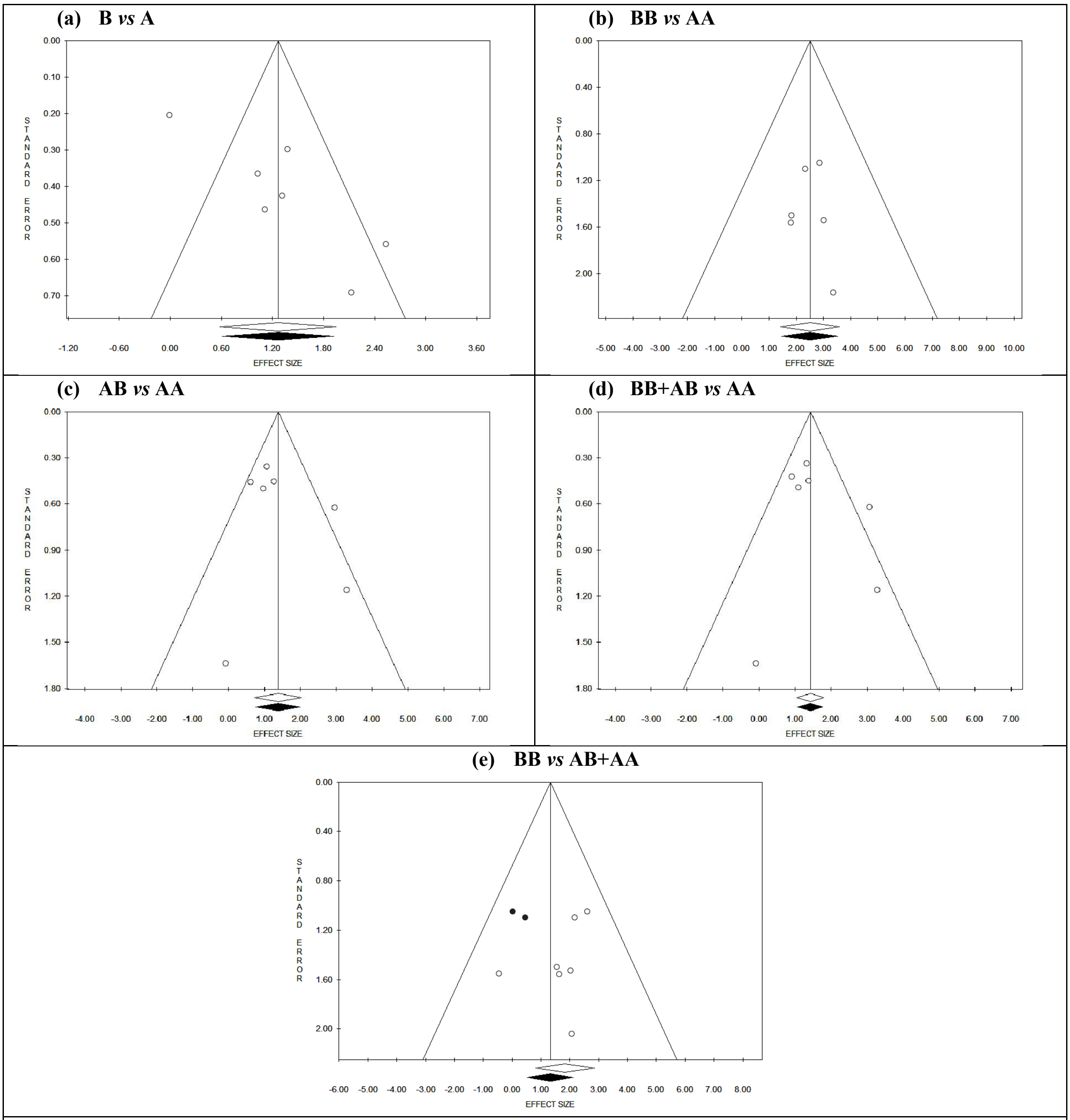

FIG. S4 | Funnel plot and trim-and-fill analysis of studies that evaluated the association between rs1800450 (A/B=G/A allele) polymorphism and RVVC. Effect Size (OR) against standard error in (a) Allelic, (b) homozygote, (c) heterozygote, (d) dominant and (e) recessive genetic models.

FIG. S5

(a) B *vs* A

	ES	95% CI	W	Sig.
Kalia et al., 2017	0.99	0.66 / 1.48	54.78%	0.962
Liu et al., 2006	8.39	2.16 / 32.56	45.22%	0.002
Asian	2.60	0.32 / 20.94	100.00%	0.368
Babula et al., 2003	12.62	4.22 / 37.75	45.58%	0.000
Donders et al., 2008	3.72	1.62 / 8.57	54.42%	0.002
Caucasian	6.49	1.97 / 21.38	100.00%	0.002
Hammad et al., 2018	3.04	1.23 / 7.55	100.00%	0.016
Egyptian	3.04	1.23 / 7.55	100.00%	0.016
Girlado et al., 2007	2.81	1.37 / 5.75	40.22%	0.005
Wojitani et al., 2012	3.98	2.21 / 7.16	59.78%	0.000
Mixed	3.46	2.20 / 5.45	100.00%	0.000

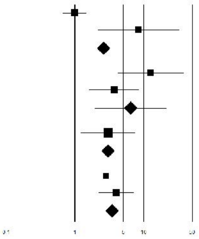

(b) BB *vs* AA

	ES	95% CI	W	Sig.
Liu et al., 2006	29.00	0.42 / 2021.75	100.00%	0.120
Asian	29.00	0.42 / 2021.75	100.00%	0.120
Babula et al., 2003	20.48	0.99 / 422.61	48.62%	0.051
Donders et al., 2008	6.28	0.33 / 119.36	51.38%	0.221
Caucasian	11.16	1.35 / 92.10	100.00%	0.025
Hammad et al., 2018	6.18	0.29 / 132.15	100.00%	0.244
Egyptian	6.18	0.29 / 132.15	100.00%	0.244
Girlado et al., 2007	10.26	1.18 / 89.21	47.74%	0.035
Wojitani et al., 2012	17.38	2.20 / 137.35	52.26%	0.007
Mixed	13.51	3.03 / 60.22	100.00%	0.001

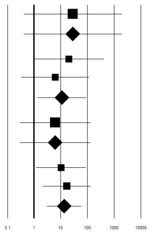

(c) AB *vs* AA

	ES	95% CI	W	Sig.
Kalia et al., 2017	0.94	0.04 / 23.25	44.06%	0.967
Liu et al., 2006	26.88	2.76 / 261.65	55.94%	0.005
Asian	6.12	0.23 / 160.65	100.00%	0.277
Babula et al., 2003	19.50	5.72 / 66.42	46.78%	0.000
Donders et al., 2008	3.58	1.47 / 8.71	53.22%	0.005
Caucasian	7.91	1.51 / 41.53	100.00%	0.015
Hammad et al., 2018	2.65	0.99 / 7.10	100.00%	0.052
Egyptian	2.65	0.99 / 7.10	100.00%	0.052
Girlado et al., 2007	1.85	0.75 / 4.56	37.93%	0.180
Wojitani et al., 2012	2.90	1.43 / 5.86	62.07%	0.003
Mixed	2.44	1.40 / 4.26	100.00%	0.002

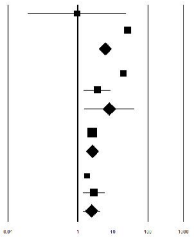

(d) BB+AB *vs* AA

	ES	95% CI	W	Sig.
Kalia et al., 2017	0.93	0.04 / 23.02	33.41%	0.963
Liu et al., 2006	26.88	2.76 / 261.65	66.59%	0.005
Asian	8.72	1.36 / 55.88	100.00%	0.022
Babula et al., 2003	21.75	6.43 / 73.62	34.42%	0.000
Donders et al., 2008	3.98	1.64 / 9.62	65.58%	0.002
Caucasian	7.14	3.49 / 14.59	100.00%	0.000
Hammad et al., 2018	3.01	1.14 / 7.93	100.00%	0.026
Egyptian	3.01	1.14 / 7.93	100.00%	0.026
Girlado et al., 2007	2.50	1.09 / 5.75	38.99%	0.031
Wojitani et al., 2012	3.80	1.95 / 7.40	61.01%	0.000
Mixed	3.23	1.92 / 5.43	100.00%	0.000

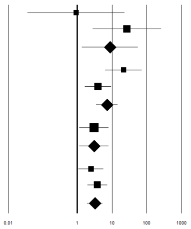

(e) BB *vs* AB+AA

	ES	95% CI	W	Sig.
Kalia et al., 2017	0.64	0.03 / 13.61	63.29%	0.778
Liu et al., 2006	7.92	0.14 / 434.29	36.71%	0.311
Asian	1.62	0.14 / 18.32	100.00%	0.697
Babula et al., 2003	7.71	0.39 / 153.96	49.08%	0.181
Donders et al., 2008	4.73	0.25 / 89.54	50.92%	0.300
Caucasian	6.01	0.74 / 49.00	100.00%	0.094
Hammad et al., 2018	5.17	0.24 / 110.12	100.00%	0.292
Egyptian	5.17	0.24 / 110.12	100.00%	0.292
Girlado et al., 2007	8.86	1.03 / 76.19	47.84%	0.047
Wojitani et al., 2012	13.50	1.72 / 105.93	52.16%	0.013
Mixed	11.04	2.49 / 48.88	100.00%	0.002

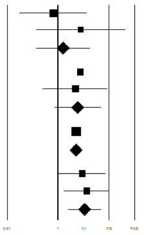

FIG. S5 | Subgroup analysis performed on the basis of ethnicity for the association between rs1800450 (A/B=G/A allele) polymorphism and RVVC under the (a) allelic, (b) homozygote, (c) heterozygote, (d) dominant and (e) recessive mode of inheritance for the studies including Babula *et al*., 2003 [8]; Donders *et al*., 2008 [18]; Girlado *et al*., 2007 [17]; Hammad *et al*., 2018 [21]; Kalia *et al*., 2017 [22]; Liu *et al*., 2006 [9] and Wojitani *et al*., 2012 [19]. ES, effect size (OR); Sig, statistical significance.

FIG. S6

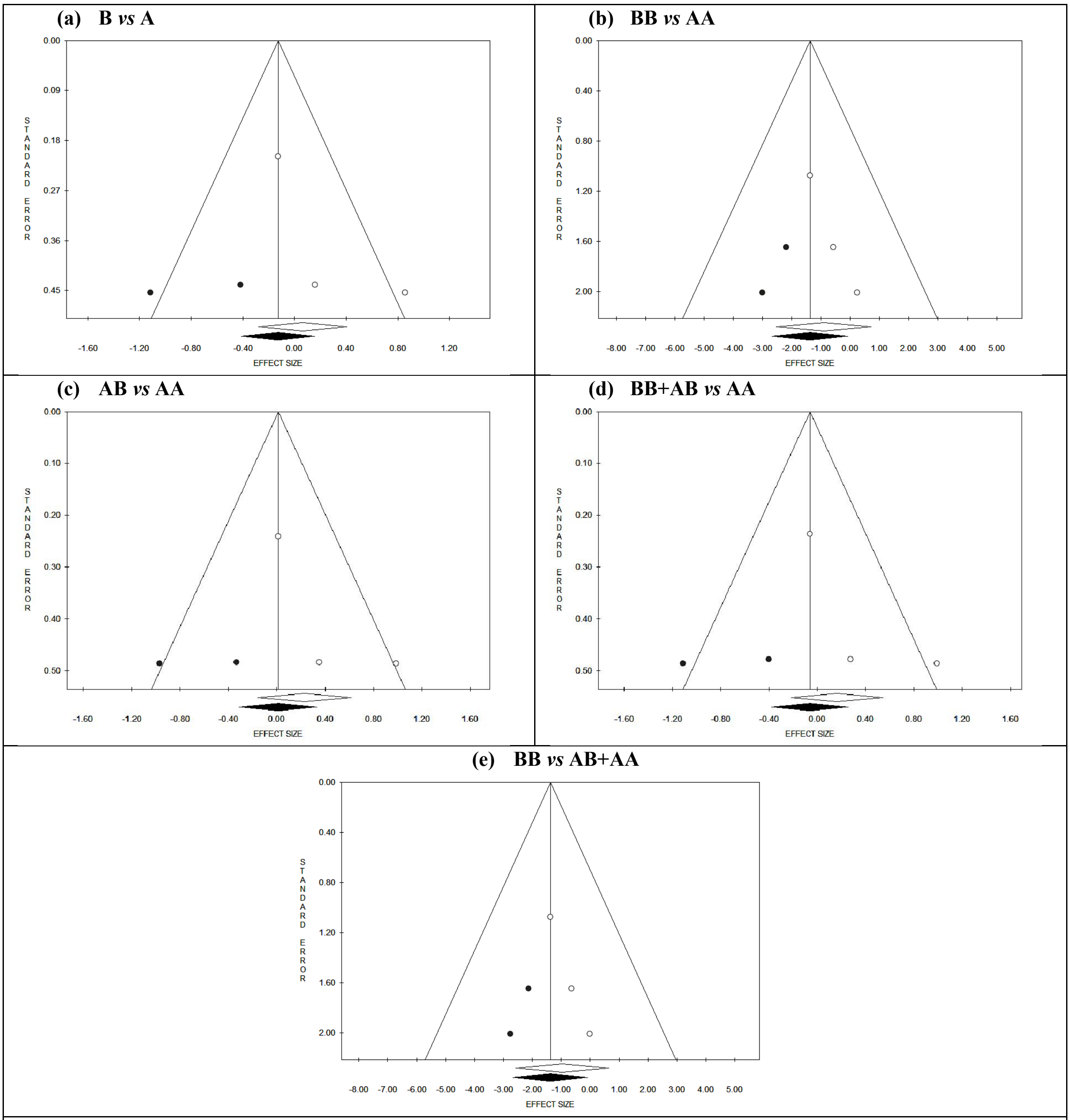

FIG. S6 | Funnel plot and trim-and-fill analysis of studies that evaluated the association between rs1800450 (A/B=G/A allele) polymorphism and VVI. Effect Size (OR) against standard error in (a) Allelic, (b) homozygote, (c) heterozygote, (d) dominant and (e) recessive genetic models.

FIG. S7

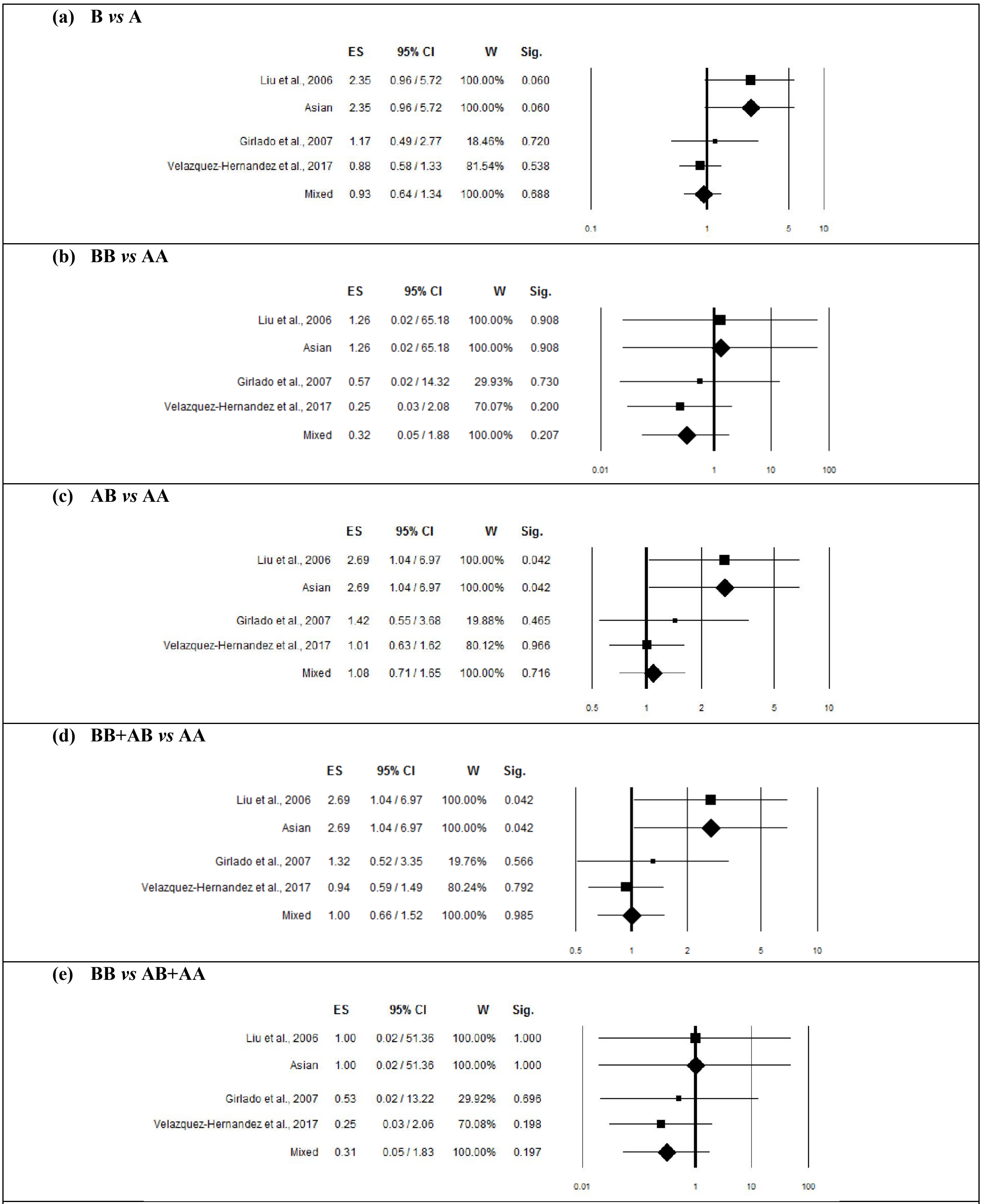

FIG. S7 | Subgroup analysis performed on the basis of ethnicity for the association between rs1800450 (A/B=G/A allele) polymorphism and VVI under the (a) allelic, (b) homozygote, (c) heterozygote, (d) dominant and (e) recessive mode of inheritance for the studies including Girlado *et al.*, 2007 [17]; Liu *et al.*, 2006 [9] and Velazquez-Hernandez *et al.*, 2017 [20]. ES, effect size (OR); Sig, statistical significance.

FIG. S8

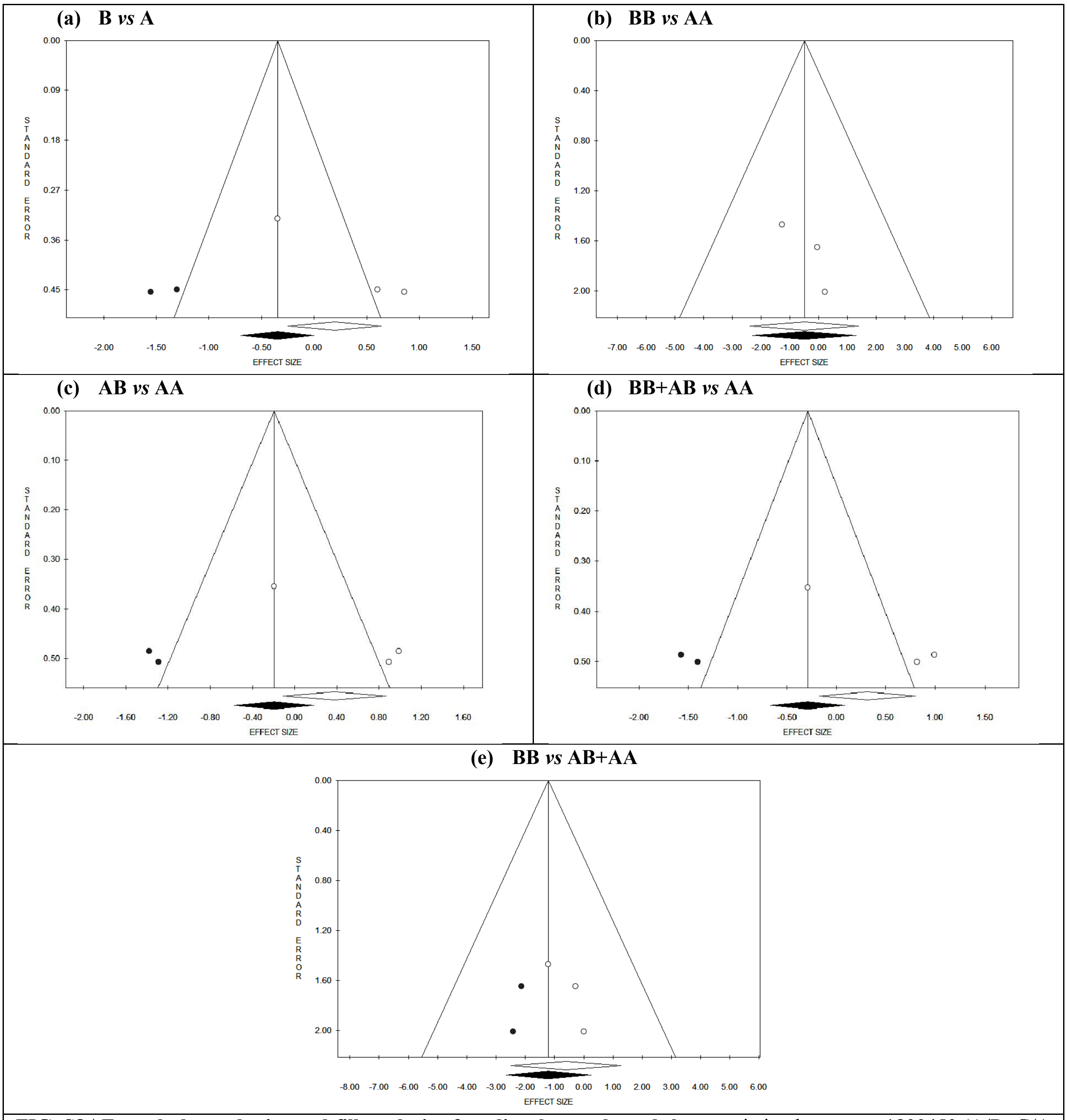

FIG. S8 | Funnel plot and trim-and-fill analysis of studies that evaluated the association between rs1800450 (A/B=G/A allele) polymorphism and VVC. Effect Size (OR) against standard error in (a) Allelic, (b) homozygote, (c) heterozygote, (d) dominant and (e) recessive genetic models.

FIG. S9

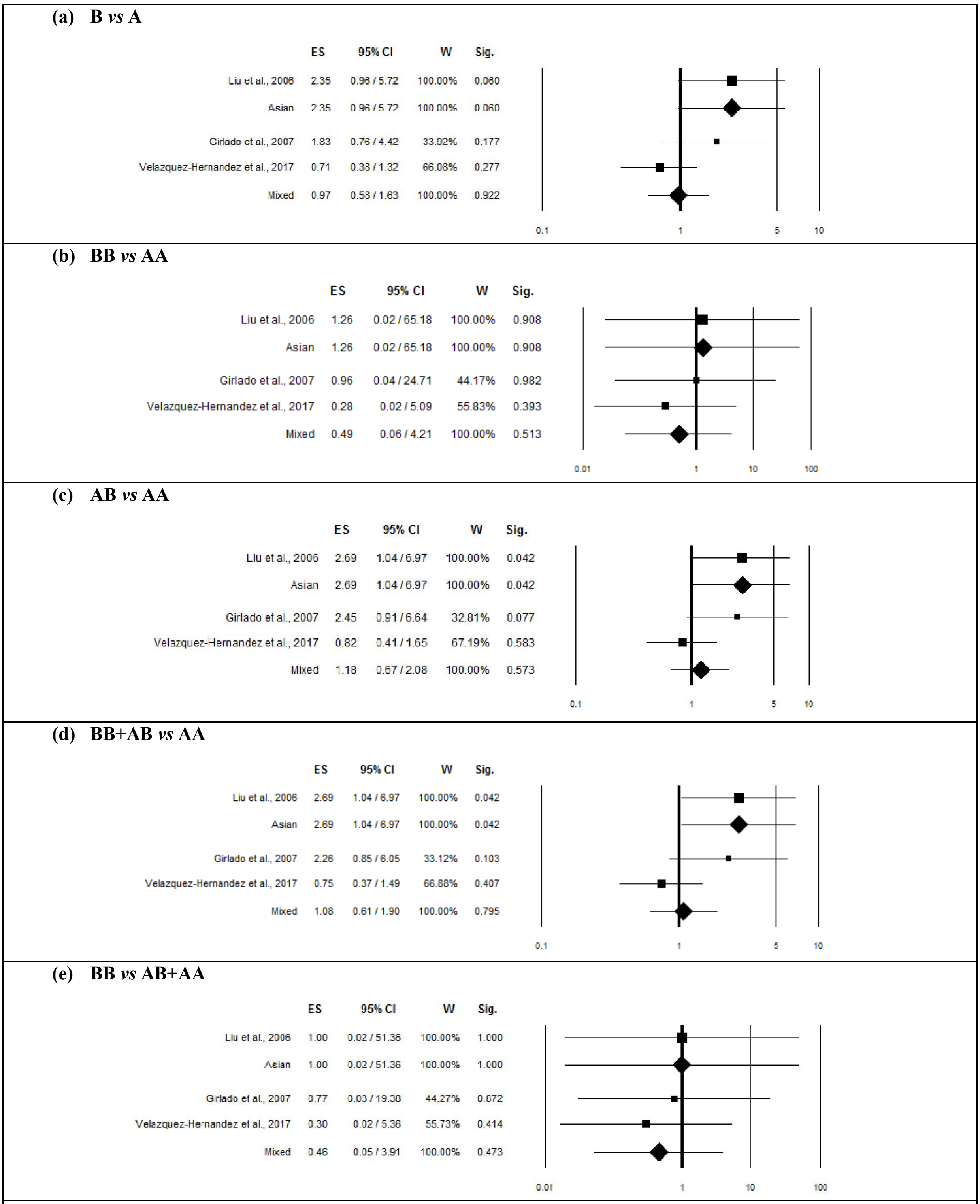

FIG. S9 | Subgroup analysis performed on the basis of ethnicity for the association between rs1800450 (A/B=G/A allele) polymorphism and VVC under the (a) allelic, (b) homozygote, (c) heterozygote, (d) dominant and (e) recessive mode of inheritance for the studies including Girlado *et al.*, 2007 [17]; Liu *et al.*, 2006 [9] and Velazquez-Hernandez *et al.*, 2017 [20]. ES, effect size (OR); Sig, statistical significance.

FIG. S10

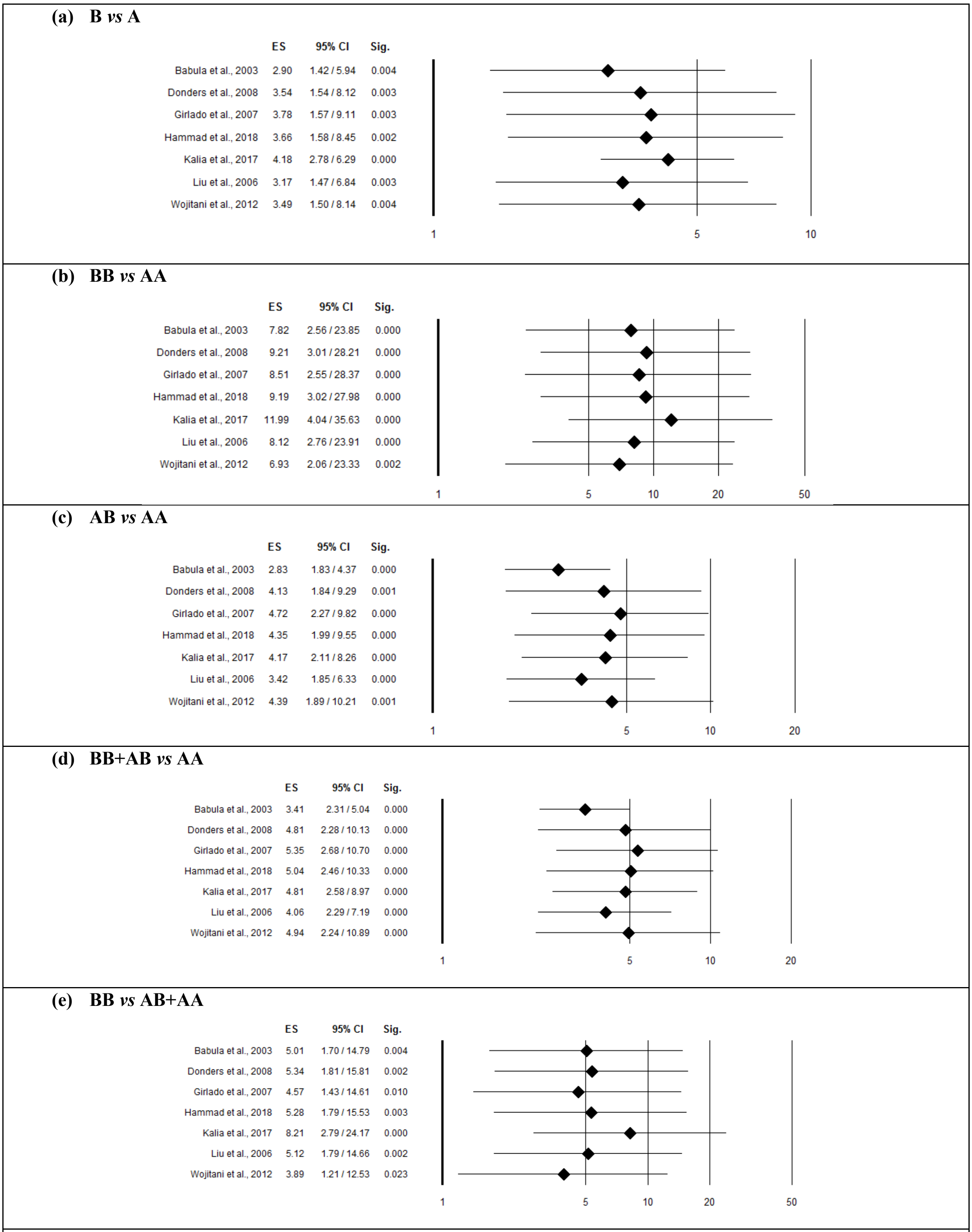

FIG. S10 | Sensitivity analysis of the association between rs1800450 (A/B=G/A allele) polymorphism and RVVI susceptibility, by excluding individual studies one at a time and recalculating the pooled odds ratio (OR) estimates for the remaining studies. (a) Allelic, (b) homozygote, (c) heterozygote, (d) dominant and (e) recessive genetic contrast for the studies including Babula *et al.*, 2003 [8]; Donders *et al.*, 2008 [18]; Girlado *et al.*, 2007 [17]; Hammad *et al.*, 2018 [21]; Kalia *et al.*, 2017 [22]; Liu *et al.*, 2006 [9] and Wojitani *et al.*, 2012 [19]. ES, effect size (OR); Sig, statistical significance.

FIG. S11

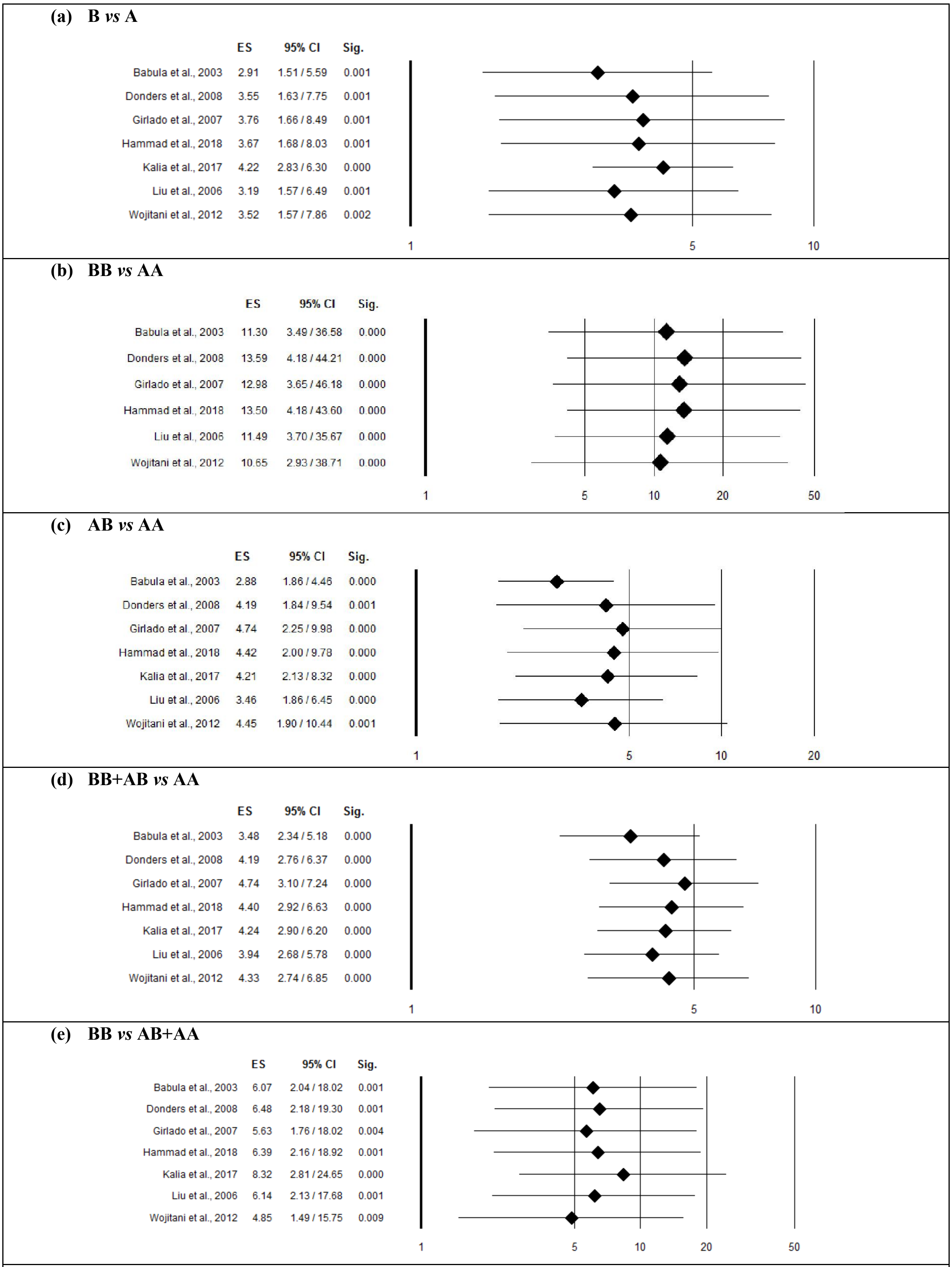

FIG. S11 | Sensitivity analysis of the association between rs1800450 (A/B=G/A allele) polymorphism and RVVC susceptibility, by excluding individual studies one at a time and recalculating the pooled odds ratio (OR) estimates for the remaining studies. (a) Allelic, (b) homozygote, (c) heterozygote, (d) dominant and (e) recessive genetic contrast for the studies including Babula *et al.*, 2003 [8]; Donders *et al.*, 2008 [18]; Girlado *et al.*, 2007 [17]; Hammad *et al.*, 2018 [21]; Kalia *et al.*, 2017 [22]; Liu *et al.*, 2006 [9] and Wojitani *et al.*, 2012 [19]. ES, effect size (OR); Sig, statistical significance.

FIG. S12

(a) B *vs* A

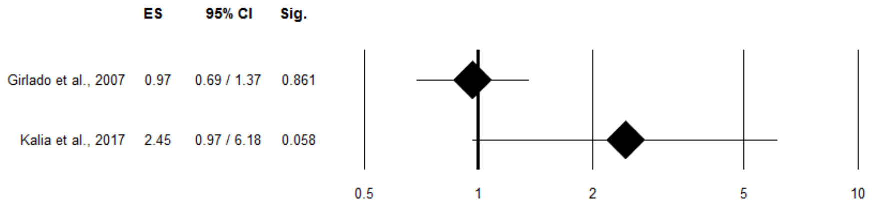

(b) BB *vs* AA

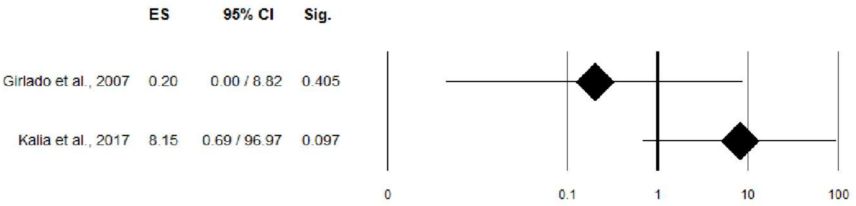

(c) AB *vs* AA

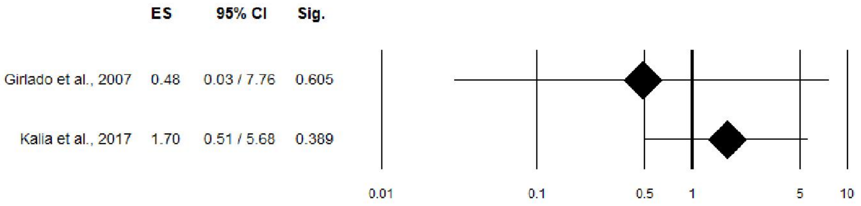

(d) BB+AB *vs* AA

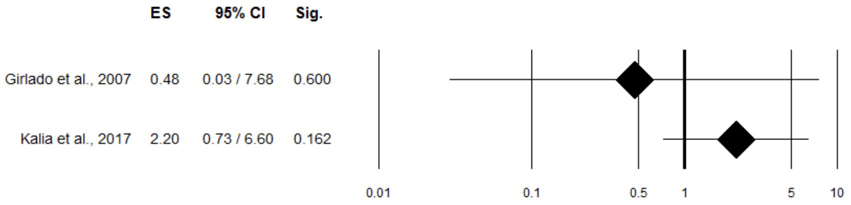

(e) BB *vs* AB+AA

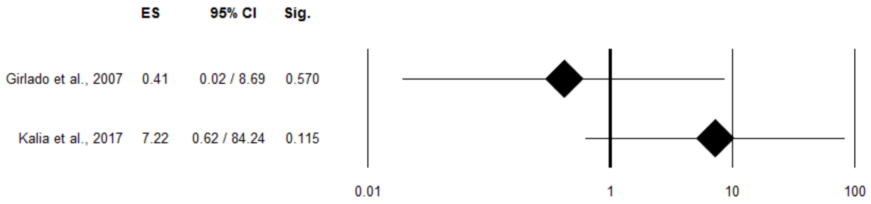

FIG. S12 | Sensitivity analysis of the association between rs1800450 (A/B=G/A allele) polymorphism and RBV susceptibility, by excluding individual studies one at a time and recalculating the pooled odds ratio (OR) estimates for the remaining studies. (a) Allelic, (b) homozygote, (c) heterozygote, (d) dominant and (e) recessive genetic contrast for the studies including Girlado *et al*., 2007 [17] and Kalia *et al*., 2017 [22]. ES, effect size (OR); Sig, statistical significance.

FIG. S13

(a) B *vs* A

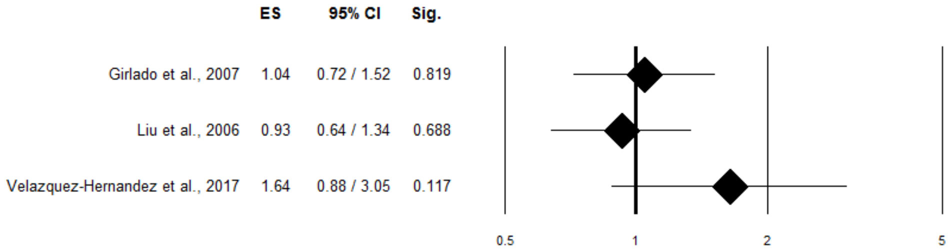

(b) BB *vs* AA

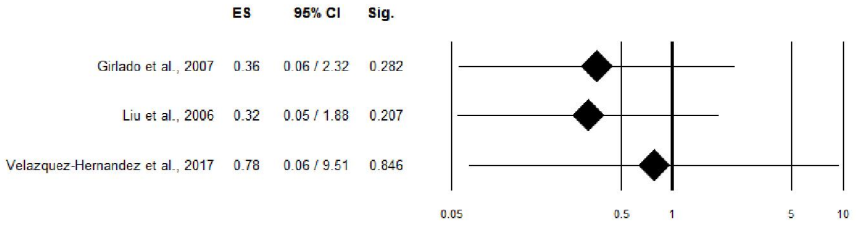

(c) AB *vs* AA

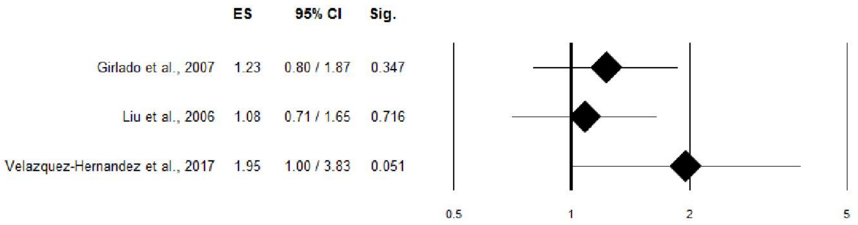

(d) BB+AB *vs* AA

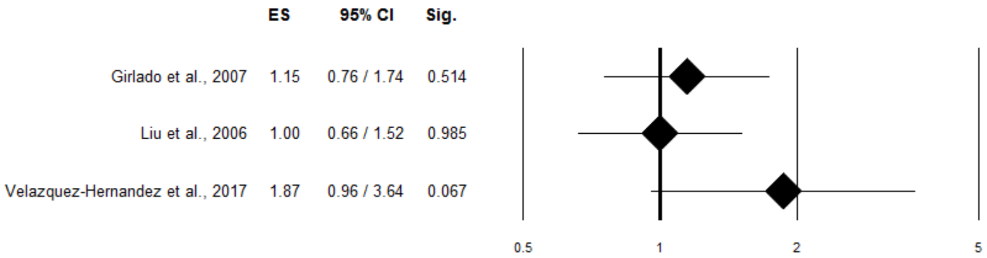

(e) BB *vs* AB+AA

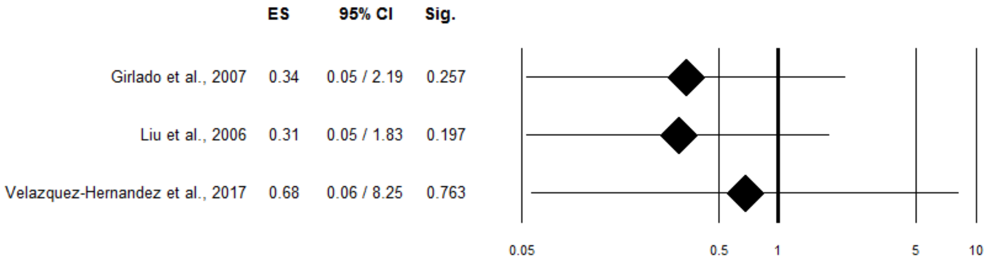

FIG. S13 | Sensitivity analysis of the association between rs1800450 (A/B=G/A allele) polymorphism and VVI susceptibility, by excluding individual studies one at a time and recalculating the pooled odds ratio (OR) estimates for the remaining studies. (a) Allelic, (b) homozygote, (c) heterozygote, (d) dominant and (e) recessive genetic contrast for the studies including Girlado *et al.*, 2007 [17]; Liu *et al.*, 2006 [9] and Velazquez-Hernandez *et al.*, 2017 [20]. ES, effect size (OR); Sig, statistical significance.

FIG. S14

(a) B *vs* A

	ES	95% CI	Sig.
Girlado et al., 2007	1.05	0.63 / 1.76	0.840
Liu et al., 2006	0.97	0.58 / 1.63	0.922
Velazquez-Hernandez et al., 2017	2.07	1.11 / 3.87	0.022

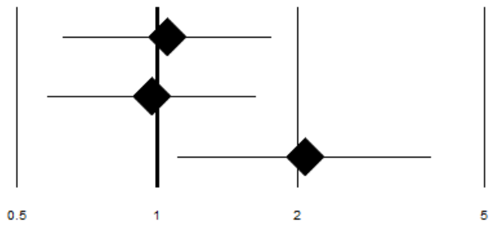

(b) BB *vs* AA

	ES	95% CI	Sig.
Girlado et al., 2007	0.48	0.05 / 4.90	0.534
Liu et al., 2006	0.49	0.06 / 4.21	0.513
Velazquez-Hernandez et al., 2017	1.07	0.09 / 13.16	0.955

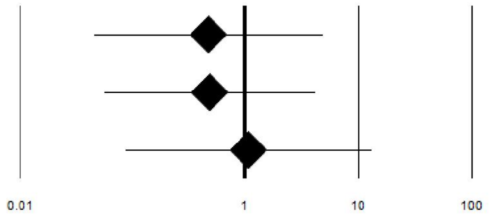

(c) AB *vs* AA

	ES	95% CI	Sig.
Girlado et al., 2007	1.24	0.71 / 2.18	0.450
Liu et al., 2006	1.18	0.67 / 2.08	0.573
Velazquez-Hernandez et al., 2017	2.57	1.29 / 5.12	0.007

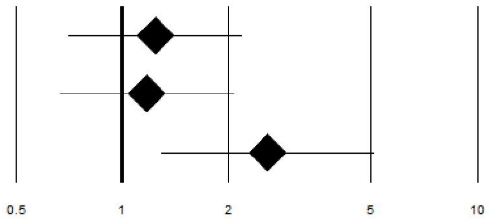

(d) BB+AB *vs* AA

	ES	95% CI	Sig.
Girlado et al., 2007	1.16	0.66 / 2.03	0.601
Liu et al., 2006	1.08	0.61 / 1.90	0.795
Velazquez-Hernandez et al., 2017	2.47	1.25 / 4.90	0.009

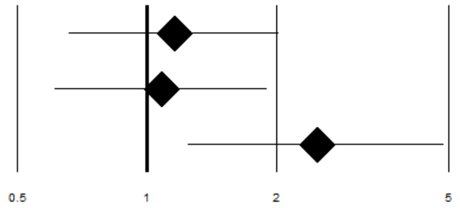

(e) BB *vs* AB+AA

	ES	95% CI	Sig.
Girlado et al., 2007	0.46	0.04 / 4.67	0.509
Liu et al., 2006	0.46	0.05 / 3.91	0.473
Velazquez-Hernandez et al., 2017	0.85	0.07 / 10.37	0.901

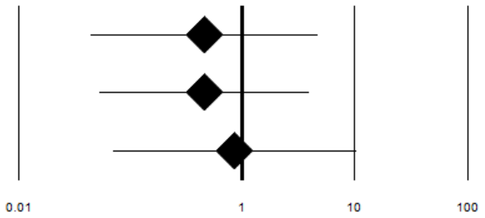

FIG. S14 | Sensitivity analysis of the association between rs1800450 (A/B=G/A allele) polymorphism and VVC susceptibility, by excluding individual studies one at a time and recalculating the pooled odds ratio (OR) estimates for the remaining studies. (a) Allelic, (b) homozygote, (c) heterozygote, (d) dominant and (e) recessive genetic contrast for the studies including Girlado *et al.*, 2007 [17]; Liu *et al.*, 2006 [9] and Velazquez-Hernandez *et al.*, 2017 [20]. ES, effect size (OR); Sig, statistical significance.

FIG. S15

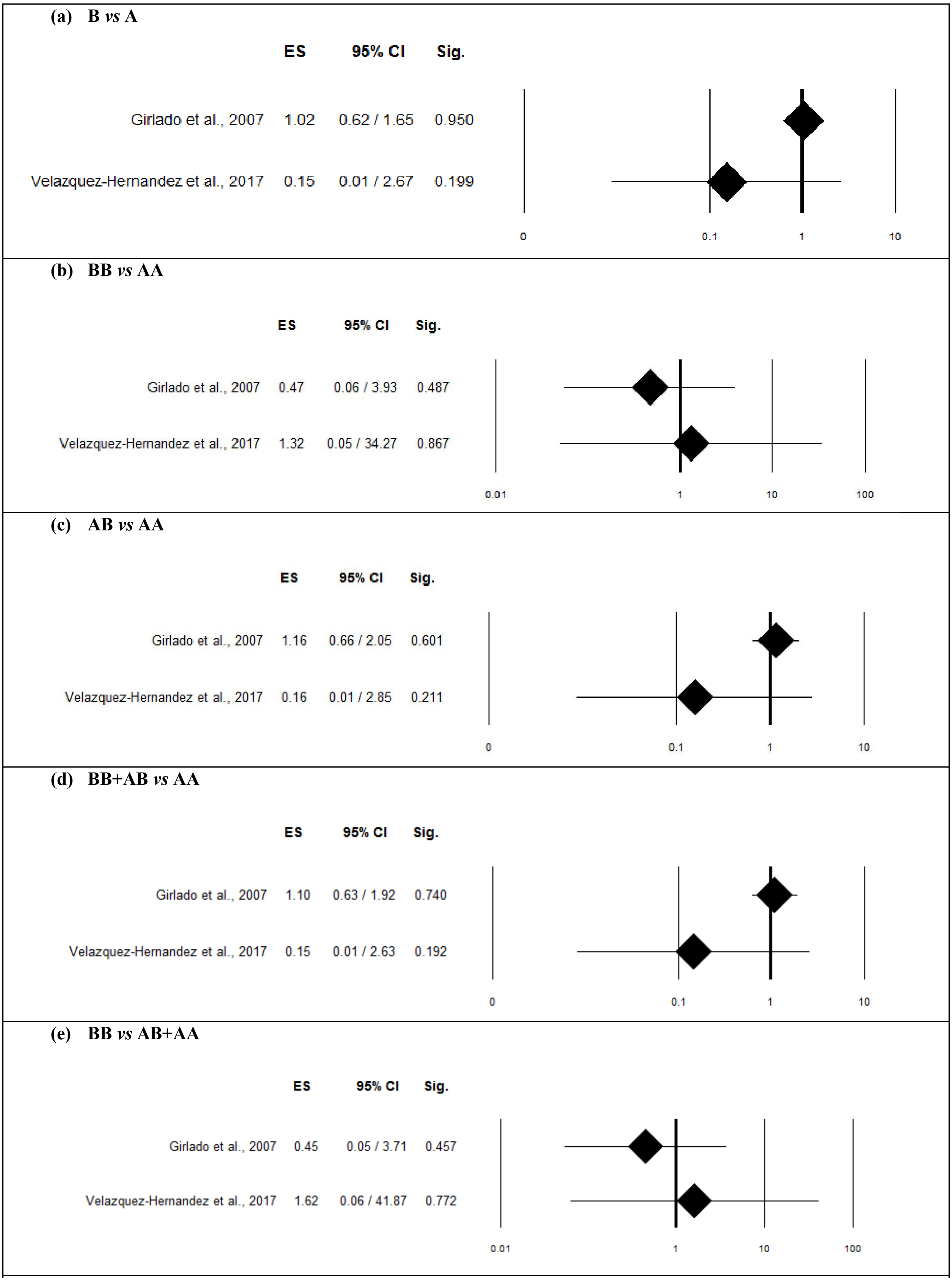

FIG. S15 | Sensitivity analysis of the association between rs1800450 (A/B=G/A allele) polymorphism and BV susceptibility, by excluding individual studies one at a time and recalculating the pooled odds ratio (OR) estimates for the remaining studies. (a) Allelic, (b) homozygote, (c) heterozygote, (d) dominant and (e) recessive genetic contrast the studies including Girlado *et al*., 2007 [17] and Velazquez-Hernandez *et al*., 2017 [20]. ES, effect size (OR); Sig, statistical significance.

FIG. S16

(a) O *vs* A

	ES	95% CI	Sig.
De Seta et al., 2007	0.94	0.64 / 1.37	0.751
Milanese et al., 2008	0.99	0.61 / 1.62	0.971

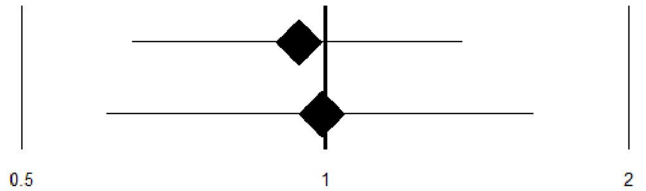

(b) OO *vs* AA

	ES	95% CI	Sig.
De Seta et al., 2007	1.37	0.50 / 3.75	0.536
Milanese et al., 2008	0.54	0.11 / 2.74	0.460

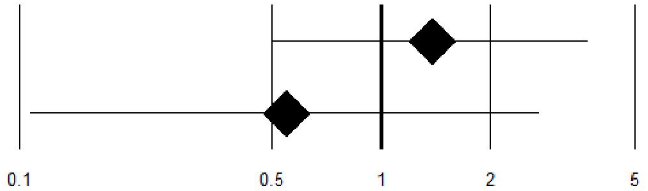

(c) AO *vs* AA

	ES	95% CI	Sig.
De Seta et al., 2007	0.78	0.48 / 1.25	0.296
Milanese et al., 2008	1.18	0.65 / 2.17	0.584

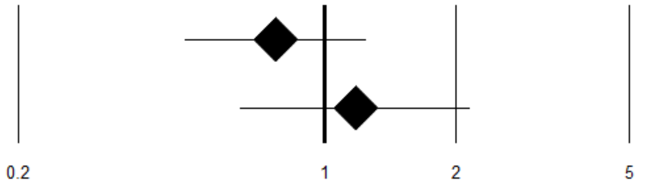

(d) OO+AO *vs* AA

	ES	95% CI	Sig.
De Seta et al., 2007	0.84	0.53 / 1.32	0.453
Milanese et al., 2008	1.10	0.61 / 1.97	0.756

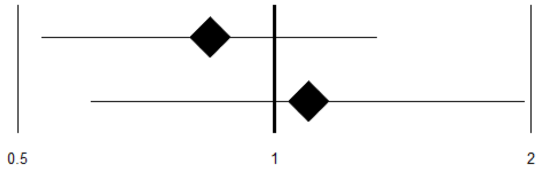

(e) OO *vs* AO+AA

	ES	95% CI	Sig.
De Seta et al., 2007	1.50	0.55 / 4.03	0.426
Milanese et al., 2008	0.51	0.10 / 2.52	0.408

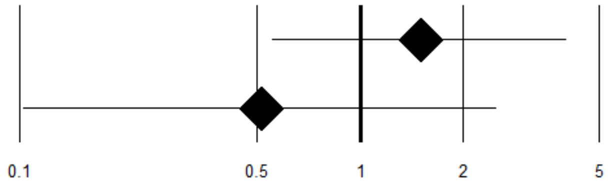

FIG. S16 | Sensitivity analysis of the association between combined Exon1 polymorphisms (involving A/O allele = C,G,G/T,A,A alleles of rs5030737, rs1800450 and rs1800451 polymorphisms respectively) and RVVI susceptibility, by excluding individual studies one at a time and recalculating the pooled odds ratio (OR) estimates for the remaining studies. (a) Allelic, (b) homozygote, (c) heterozygote, (d) dominant and (e) recessive genetic contrast for the studies including De Seta *et al*., 2007 [16] and Milanese *et al*., 2008 [10]. ES, effect size (OR); Sig, statistical significance.

FIG. S17

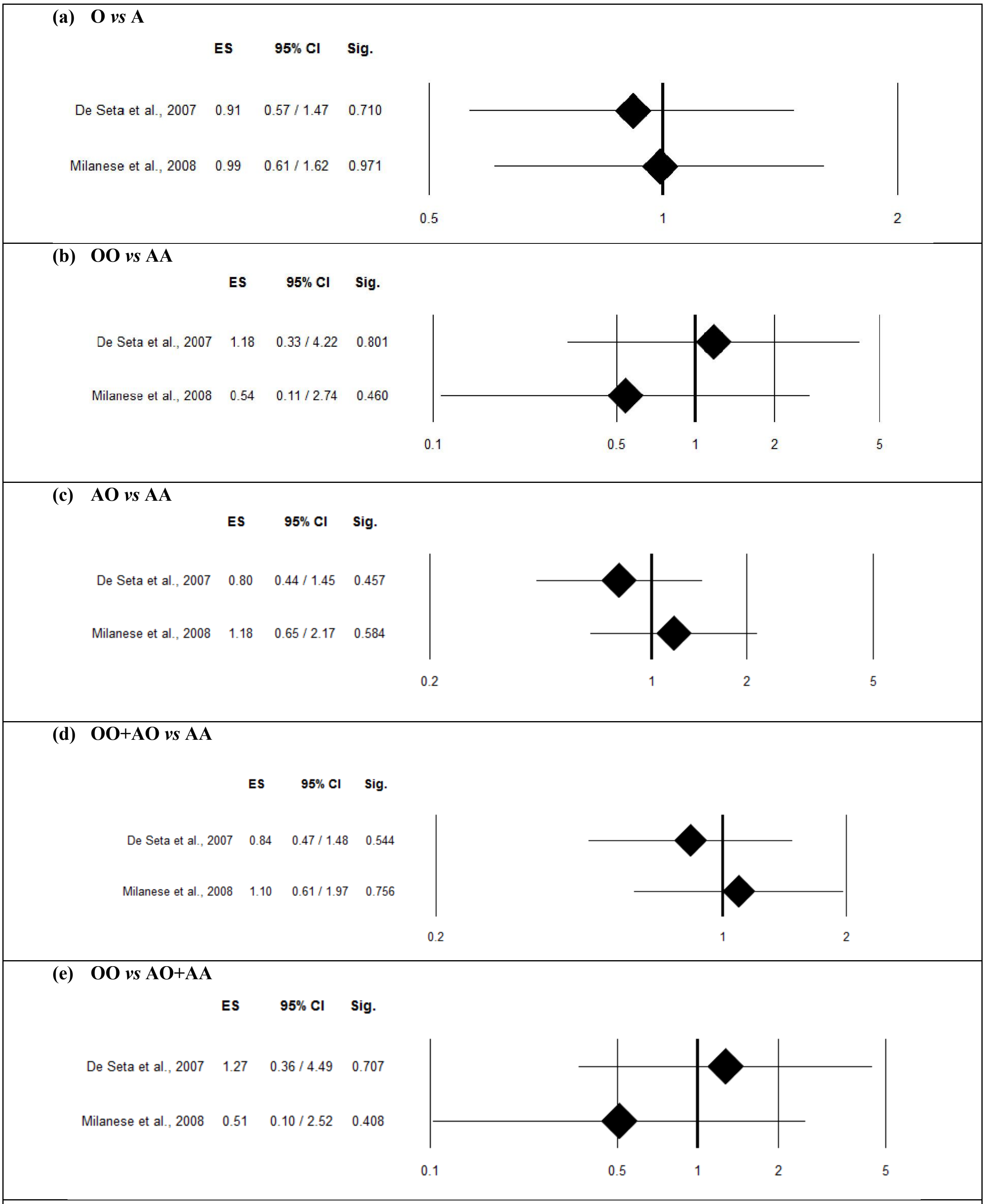

FIG. S17 | Sensitivity analysis of the association between combined Exon1 polymorphisms (involving A/O allele = C,G,G/T,A,A alleles of rs5030737, rs1800450 and rs1800451 polymorphisms respectively) and RBV susceptibility, by excluding individual studies one at a time and recalculating the pooled odds ratio (OR) estimates for the remaining studies. (a) Allelic, (b) homozygote, (c) heterozygote, (d) dominant and (e) recessive genetic contrast for the studies including De Seta *et al*., 2007 [16] and Milanese *et al*., 2008 [10]. ES, effect size (OR); Sig, statistical significance.

SUPPLEMENTARY TABLES

Table S1 Genotypic and allelic distribution of *MBL2* gene polymorphisms included in this meta-analysis.

SNPs & related studies	Cases						Controls					§HWE (p-value)
	Cases types	Genotypes			Alleles		Genotypes			Alleles		
**rs1800450**		**AA**	**AB**	**BB**	**A**	**B**	**AA**	**AB**	**BB**	**A**	**B**	
Babula *et al.* [8]	RVVC	13	26	3	52	32	39	4	0	82	4	0.749
Liu *et al.* [9]	RVVC	1	5	0	7	5	43	8	0	94	8	0.543
	VVC	34	17	0	85	17						
Girlado *et al.* [17]	RVVC	31	13	6	75	25	53	12	1	118	14	0.750
	RBV	13	5	2	31	9						
	‡RVVI (RVVC+RBV)	44	18	8	106	34						
	VVC	18	10	0	46	10						
	BV	13	0	0	26	0						
	‡VVI (BV+VVC)	31	10	0	72	10						
Donders *et al.* [18]	RVVC	69	36	4	174	44	48	7	0	103	7	0.614
Wojitani *et al.* [19]	RVVC	58	30	12	146	54	84	15	1	183	17	0.721
Velazquez-Hernandez *et al.* [20]	VVC	36	13	0	85	13	155	68	7	378	82	0.889
	BV	47	24	1	118	26						
	TV	5	2	0	12	2						
	†VVI	88	39	1	215	41						
Hammad *et al.* [21]	RVVC	42	15	2	99	19	52	7	0	111	7	0.628
Kalia *et al.* [22]	RVVC	0	62	0	62	62	1	200	2	202	204	0.000
	RBV	1	96	0	98	96						
	MI	0	41	0	41	41						
	†RVVI	1	257	0	259	257						
**rs1800451**		**AA**	**AC**	**CC**	**A**	**C**	**AA**	**AC**	**CC**	**A**	**C**	
Girlado *et al.* [17]*	RVVC	-	2	-	-	-	63	2	1	128	4	0.000
	RBV	-	3	-	-	-						
Velazquez-Hernandez *et al.* [20]	VVC	47	2	0	96	2	217	13	0	447	13	0.659
	BV	67	5	0	139	5						
	TV	7	0	0	14	0						
	†VVI	121	7	0	249	7						
**Combined Exon 1 (rs5030737, rs1800450 and rs1800451)**		**AA**	**AO**	**OO**	**A**	**O**	**AA**	**AO**	**OO**	**A**	**O**	
De Seta *et al.* [16]	RBV	41	28	2	110	32	78	45	7	201	59	0.879
Milanese *et al.* [10]	RBV	48	22	4	118	30	99	57	7	255	71	0.737
	RVVC	55	24	6	134	36						
	‡ RVVI (RBV+RVVC)	103	46	10	252	66						
**SNPs including rs11003125, rs7096206, rs7095891** was evaluated by single study *i.e.* Kalia *et al.*, 2017 [22], **rs10824792, rs2120132, rs2120131, rs2165813, rs2099903 and rs2099902** was evaluated by single study *i.e.* Kalia *et al.*, 2019 [23], **rs11003124, rs7084554, rs36014597 and rs11003123** was evaluated by single study *i.e.* Kalia *et al.*, 2019b [24].												

Note: † reported genetic distribution of RVVI/VVI, ‡ Self calculated distribution of RVVI/VVI, § Hardy Weinberg equilibrium of controls, * the genotype information for rs1800451 polymorphism could not be obtained even after repeat contacts to authors of the study. rs1800450 (Codon 54, A/B (G>A transition)), rs1800451 (Codon 57, A/C (G>A transition) and combined Exon1 polymorphisms (involving A/O allele = C,G,G/T,A,A alleles of rs5030737, rs1800450 and rs1800451 polymorphisms respectively)

Table S2 Quality assessment according to the Newcastle-Ottawa Scale for the included studies

	Selection				Comparability	Exposure		
**Studies**	**Adequacy of case definition**	**Representativeness of cases**	**Selection of controls**	**Definition of controls**	**Comparability of cases and controls**	**Ascertainment of exposure**	**Same method of ascertainment for cases and controls**	**Rate of Non-response**
Babula *et al*. [8]	*	*	*	*	*	*	*	*
Liu *et al*., [9]	*	*	*	*	*	*	*	*
De Seta *et al*.[16]	*	*	*	*	*	*	*	*
Girlado *et al*. [17]	*	*	*	*	*	*	*	*
Donders *et al*. [18]	*	*	*	*	*	*	*	*
Milanese *et al*. [10]	*	*	*	*	-	*	*	*
Wojitani *et al*. [19]	*	*	*	*	*	*	*	*
Velazquez-Hernandez *et al*. [20]	*	*	*	*	-	*	*	*
Hammad *et al*. [21]	*	*	*	*	*	*	-	*
Kalia *et al*. [22]	*	*	*	*	*	*	*	*
Kalia *et al*. [23]	*	*	*	*	*	*	*	*
Kalia *et al*. [24]	*	*	*	*	*	*	*	*

Table S3 Statistics to test the publication bias and heterogeneity in the meta-analysis for rs1800450 polymorphism and RVVI susceptibility

**Comparisons** (A/B = G/A)	**Egger's regression analysis**			**Begg and Mazumdar's rank correlation test**		**Heterogeneity analysis**			**Effect Model**
	**Intercept**	**t**	**p-value**	**Z**	**p-value**	**Q-value**	$P_{heterogeneity}$	I^2 **(%)**	
B *vs* A	4.74	5.96	0.002	1.05	0.293	50.10	< 0.0001	88.02	Random
BB *vs* AA	-1.37	-0.96	0.380	-1.35	0.176	4.96	0.549	0.00	Fixed
AB *vs* AA	1.60	1.03	0.348	0.45	0.652	14.11	0.028	57.49	Random
BB+AB *vs* AA	1.31	0.91	0.406	0.45	0.652	12.74	0.047	52.91	Random
BB *vs* AB+AA	-1.79	-0.99	0.367	-1.35	0.176	6.24	0.397	3.85	Fixed

Table S4 Trim and fill analysis for adjustment of publication bias in the meta-analysis for rs1800450 polymorphism and RVVI susceptibility

**Comparisons** (A/B = G/A)	**Overall effect size (observed)**			**Overall effect size (estimated)**			**Trimmed studies**	p_c
	**OR**	**CI**	**p-value**	**OR**	**CI**	**p-value**		
B *vs* A	**3.54**	1.69-7.43	0.001	**3.54**	1.69-7.43	0.001	0	0.005
BB *vs* AA	**8.78**	3.08-24.98	0.000047	**8.12**	2.94-22.43	5.3×10^{-5}	1	2.6×10^{-4}
AB *vs* AA	**3.92**	2.05-7.50	0.00004	**3.92**	2.05-7.50	4.0×10^{-5}	0	2.0×10^{-4}
BB+AB *vs* AA	**4.54**	2.50-8.24	6.9×10^{-7}	**4.54**	2.50-8.24	6.9×10^{-7}	0	3.2×10^{-6}
BB *vs* AB+AA	**5.27**	1.90-14.57	0.001	**2.76**	1.19-6.37	0.018	2	0.09

OR= Odds ratio, CI = 95% confidence intervals, p_c = p-value after Bonferroni correction (estimated P value × 5 genetic models), Bold numbers indicates statistically significant OR values.

Table S5 Moderator and subgroup analysis: rs1800450 polymorphism and RVVI susceptibility

Ethnicity	K	N	ES	95% CI	P	p_c	Q	I^2	p[a]
**B *vs* A**									0.745
Asian	2	1036	2.58	0.32-20.88	0.375	1.5	9.27**	89.22	
Caucasian	2	498	**6.49**	1.97-21.38	0.002	0.008	3.02	66.88	
Egyptian	1	236	3.04	1.23-7.55	0.016	0.064	-	-	
Mixed	2	672	**3.37**	2.16-5.25	0.00001	0.00004	0.72	0.00	
**BB *vs* AA**									0.897
Asian	2	48	2.19	0.02-286.09	0.753	3.012	2.94	65.99	
Caucasian	2	176	11.16	1.35-92.10	0.025	0.1	0.30	0.00	
Egyptian	1	96	6.18	0.29-132.15	0.244	0.976	-	-	
Mixed	2	261	**13.03**	2.94-57.19	0.001	0.004	0.15	0.00	
**AB *vs* AA**									0.538
Asian	2	516	6.55	0.34-127.94	0.215	0.086	2.75	63.68	
Caucasian	2	242	7.91	1.51-41.53	0.015	0.06	4.82*	79.24	
Egyptian	1	116	2.65	0.99-7.10	0.052	0.208	-	-	
Mixed	2	314	**2.38**	1.39-4.07	0.002	0.008	0.72	0.00	
**BB+AB *vs* AA**									0.658
Asian	2	518	6.52	0.33-128.52	0.218	0.872	2.77	63.92	
Caucasian	2	249	**8.81**	1.67-46.43	0.010	0.04	4.89*	79.57	
Egyptian	1	118	3.01	1.14-7.93	0.026	0.104	-	-	
Mixed	2	336	**3.13**	1.89-5.19	0.00001	0.00004	0.76	0.00	
**BB *vs* AB+AA**									0.291
Asian	2	518	0.66	0.06-7.40	0.733	2.932	2.34	57.35	
Caucasian	2	249	6.01	0.74-49.00	0.094	0.376	0.05	0.00	
Egyptian	1	118	5.17	0.24-110.12	0.292	1.168	-	-	
Mixed	2	336	**10.70**	2.45-46.68	0.002	0.008	0.10	0.00	

K= Total number of studies, N= Total number of participants, ES= Effect Size, CI= Confidence Intervals, A/B allele= G/A allele, * $P_{heterogeneity}$ <0.05, ** $P_{heterogeneity}$ <0.01, *** $P_{heterogeneity}$ <0.001, p_c = p-value after Bonferroni correction (P×4 ethnic groups), 'a' p-value for comparison between subgroups, Bold numbers indicates statistically significant ES after Bonferroni correction.

Table S6 Statistics to test the publication bias and heterogeneity in the meta-analysis for rs1800450 polymorphism and RVVC susceptibility

Comparisons (A/B = G/A)	Egger's regression analysis			Begg and Mazumdar's rank correlation test		Heterogeneity analysis			Effect Model
	Intercept	t	p-value	Z	p-value	Q-value	$P_{heterogeneity}$	I^2 (%)	
B *vs* A	5.22	3.86	0.012	1.35	0.176	34.93	< 0.0001	82.82	Random
BB *vs* AA	0.10	0.12	0.910	0.19	0.851	0.79	0.977	0.00	Fixed
AB *vs* AA	1.29	0.84	0.441	0.45	0.652	13.77	0.032	56.44	Random
BB+AB *vs* AA	1.06	0.74	0.493	0.45	0.652	12.44	0.053	51.77	Fixed
BB *vs* AB+AA	-1.47	-1.24	0.270	-1.35	0.176	2.85	0.827	0.00	Fixed

Table S7 Trim and fill analysis for adjustment of publication bias in the meta-analysis for rs1800450 polymorphism and RVVC susceptibility

Comparisons (A/B = G/A)	Overall effect size (observed)			Overall effect size (estimated)			Trimmed studies	p_c
	**OR**	**CI**	**p-value**	**OR**	**CI**	**p-value**		
B *vs* A	**3.54**	1.79-7.00	0.0002	**3.54**	1.79-7.00	2.0×10^{-4}	0	0.001
BB *vs* AA	**12.22**	4.09-36.51	7.4×10^{-6}	**12.22**	4.09-36.51	7.4×10^{-6}	0	3.7×10^{-5}
AB *vs* AA	**3.97**	2.06-7.64	3.7×10^{-5}	**3.97**	2.06-7.64	3.7×10^{-5}	0	1.8×10^{-4}
BB+AB *vs* AA	**4.15**	2.85-6.06	1.5×10^{-13}	**4.15**	2.85-6.06	1.5×10^{-13}	0	7.5×10^{-13}
BB *vs* AB+AA	**6.24**	2.24-17.36	0.00045	**3.74**	1.61-8.70	0.002	2	0.01

p_c = p-value after Bonferroni correction (estimated P value × 5 genetic models), Bold numbers indicates statistically significant OR values.

Table S8 Moderator and subgroup analysis: rs1800450 polymorphism and RVVC susceptibility

**Ethnicity**	**K**	**N**	**ES**	**95% CI**	**P**	p_c	**Q**	I^2	p^a
**B *vs* A**									0.755
Asian	2	644	2.60	0.32-20.94	0.368	1.472	8.77**	88.60	
Caucasian	2	498	**6.49**	1.97-21.38	0.002	0.008	3.02	66.88	
Egyptian	1	236	3.04	1.23-7.55	0.016	0.064	-	-	
Mixed	2	632	**3.46**	2.20-5.45	0.00001	0.0004	0.55	0.00	
**BB *vs* AA**									0.945
Asian	1	44	29.00	0.42-2021.75	0.120	0.48	-	-	
Caucasian	2	176	11.16	1.35-92.10	0.025	0.1	0.30	0.00	
Egyptian	1	96	6.18	0.29-132.15	0.244	0.976	-	-	
Mixed	2	246	**13.51**	3.03-60.22	0.001	0.004	0.12	0.00	
**AB *vs* AA**									0.580
Asian	2	320	6.12	0.23-160.65	0.277	1.108	2.79	64.21	
Caucasian	2	242	7.91	1.51-41.53	0.015	0.06	4.82*	79.24	
Egyptian	1	116	2.65	0.99-7.10	0.052	0.208	-	-	
Mixed	2	296	**2.44**	1.40-4.26	0.002	0.008	0.59	0.00	
**BB+AB *vs* AA**									0.247
Asian	2	322	8.72	1.36-55.88	0.022	0.088	2.81	64.42	
Caucasian	2	249	**7.14**	3.49-14.59	7.3×10^{-8}	2.9×10^{-7}	4.89*	79.57	
Egyptian	1	118	3.01	1.14-7.93	0.026	0.104	-	-	
Mixed	2	316	**3.23**	1.92-5.43	0.00001	0.00004	0.59	0.00	
**BB *vs* AB+AA**									0.622
Asian	2	322	1.62	0.14-18.32	0.697	2.788	0.95	0.00	
Caucasian	2	249	6.01	0.74-49.00	0.094	0.376	0.05	0.00	
Egyptian	1	118	5.17	0.24-110.12	0.292	1.168	-	-	
Mixed	2	316	**11.04**	2.49-48.88	0.002	0.008	0.08	0.00	

K= Total number of studies, N= Total number of participants, ES= Effect Size, CI= Confidence Intervals, A/B allele= G/A allele, * $P_{heterogeneity}$ <0.05, ** $P_{heterogeneity}$ <0.01, *** $P_{heterogeneity}$ <0.001, p_c = p-value after Bonferroni correction (P×4 ethnic groups), 'a' p-value for comparison between subgroups Bold numbers indicates statistically significant ES after Bonferroni correction.

Table S9 Statistics to test the publication bias and heterogeneity in the meta-analysis for rs1800450 polymorphism and VVI susceptibility

**Comparisons** (A/B = G/A)	**Egger's regression analysis**			**Begg and Mazumdar's rank correlation test**		**Heterogeneity analysis**			**Effect Model**
	**Intercept**	**t**	**p-value**	**Z**	**p-value**	**Q-value**	**$P_{heterogeneity}$**	**I^2 (%)**	
B *vs* A	2.69	1.61	0.353	1.57	0.117	3.92	0.141	49.01	Fixed
BB *vs* AA	1.64	8.99	0.070	1.57	0.117	0.55	0.758	0.00	Fixed
AB *vs* AA	2.71	1.72	0.335	1.57	0.117	3.33	0.189	39.94	Fixed
BB+AB *vs* AA	2.84	1.66	0.345	1.57	0.117	3.84	0.146	47.94	Fixed
BB *vs* AB+AA	1.43	13.17	0.048	1.57	0.117	0.42	0.810	0.00	Fixed

Table S10 Trim and fill analysis for adjustment of publication bias in the meta-analysis for rs1800450 polymorphism and VVI susceptibility

**Comparisons** (A/B = G/A)	**Overall effect size (observed)**			**Overall effect size (estimated)**			**Trimmed studies**	p_c
	**OR**	**CI**	**p-value**	**OR**	**CI**	**p-value**		
B *vs* A	1.06	0.76-1.50	0.724	0.88	0.65-1.19	0.399	2	1.995
BB *vs* AA	0.40	0.08-2.02	0.270	**0.25**	0.06-0.98	0.046	2	0.23
AB *vs* AA	1.26	0.85-1.85	0.247	1.01	0.72-1.41	0.953	2	4.765
BB+AB *vs* AA	1.18	0.80-1.72	0.406	0.94	0.67-1.31	0.711	2	3.555
BB *vs* AB+AA	0.38	0.08-1.90	0.239	**0.25**	0.06-0.97	0.045	2	0.225

p_c = p-value after Bonferroni correction (estimated P value × 5 genetic models), Bold numbers indicates statistically significant OR values.

Table S11 Moderator and subgroup analysis: rs1800450 polymorphism and VVI susceptibility

**Ethnicity**	**K**	**N**	**ES**	**95% CI**	**P**	p_c	**Q**	**I^2**	**p^a**
**B *vs* A**									0.059
Asian	1	204	2.35	0.96-5.72	0.060	0.12	-	-	
Mixed	2	930	0.93	0.64-1.34	0.688	1.376	0.35	0.00	
**BB *vs* AA**									0.535
Asian	1	77	1.26	0.02-65.18	0.908	1.816	-	-	
Mixed	2	336	0.32	0.05-1.88	0.207	0.414	0.17	0.00	
**AB *vs* AA**									0.087
Asian	1	102	2.69	1.04-6.97	0.042	0.084	-	-	
Mixed	2	456	1.08	0.71-1.65	0.716	1.432	0.40	0.00	
**BB+AB *vs* AA**									0.063
Asian	1	102	2.69	1.04-6.97	0.042	0.084	-	-	
Mixed	2	465	1.00	0.66-1.52	0.985	1.97	0.40	0.00	
**BB *vs* AB+AA**									0.598
Asian	1	102	1.00	0.02-51.36	1.000	2.0	-	-	
Mixed	2	465	0.31	0.05-1.83	0.197	0.394	0.14	0.00	

K= Total number of studies, N= Total number of participants, ES= Effect Size, CI= Confidence Intervals, A/B allele= G/A allele, * $P_{heterogeneity}$ <0.05, ** $P_{heterogeneity}$ <0.01, *** $P_{heterogeneity}$ <0.001, p_c = p-value after Bonferroni correction (P×2 ethnic groups), 'a' p-value for comparison between subgroups

Table S12 Statistics to test the publication bias and heterogeneity in the meta-analysis for rs1800450 polymorphism and VVC susceptibility

Comparisons (A/B = G/A)	Egger's regression analysis			Begg and Mazumdar's rank correlation test		Heterogeneity analysis			Effect Model
	Intercept	t	p-value	Z	p-value	Q-value	$P_{heterogeneity}$	I^2 (%)	
B *vs* A	8.34	7.38	0.086	1.57	0.117	5.81	0.055	65.57	Fixed
BB *vs* AA	2.74	1.68	0.341	1.57	0.117	0.48	0.788	0.00	Fixed
AB *vs* AA	7.90	6.07	0.104	0.52	0.602	5.23	0.073	61.75	Fixed
BB+AB *vs* AA	8.40	5.59	0.113	0.52	0.602	5.89	0.053	66.04	Fixed
BB *vs* AB+AA	2.20	1.81	0.322	1.57	0.117	0.30	0.862	0.00	Fixed

Table S13 Trim and fill analysis for adjustment of publication bias in the meta-analysis for rs1800450 polymorphism and VVC susceptibility

Comparisons (A/B = G/A)	Overall effect size (observed)			Overall effect size (estimated)			Trimmed studies	p_c
	OR	CI	p-value	OR	CI	p-value		
B *vs* A	1.21	0.78-1.89	0.393	0.71	0.49-1.01	0.059	2	0.295
BB *vs* AA	0.61	0.09-4.02	0.604	0.61	0.09-4.02	0.604	0	3.02
AB *vs* AA	1.46	0.90-2.39	0.127	0.82	0.55-1.23	0.339	2	1.695
BB+AB *vs* AA	1.37	0.84-2.22	0.207	0.75	0.50-1.11	0.148	2	0.74
BB *vs* AB+AA	0.55	0.08-3.60	0.529	0.30	0.07-1.36	0.118	2	0.59

p_c = p-value after Bonferroni correction (estimated P value × 5 genetic models)

Table S14 Moderator and subgroup analysis: rs1800450 polymorphism and VVC susceptibility

Ethnicity	K	N	ES	95% CI	P	p_c	Q	I^2	p^a
**B *vs* A**									0.093
Asian	1	204	2.35	0.96-5.72	0.060	0.12	-	-	
Mixed	2	746	0.97	0.58-1.63	0.922	1.844	2.99	66.55	
**BB *vs* AA**									0.679
Asian	1	77	1.26	0.02-65.18	0.908	1.816	-	-	
Mixed	2	270	0.49	0.06-4.21	0.513	1.026	0.30	0.00	
**AB *vs* AA**									0.145
Asian	1	102	2.69	1.04-6.97	0.042	0.084	-	-	
Mixed	2	365	1.18	0.67-2.08	0.573	1.146	3.11	67.84	
**BB+AB *vs* AA**									0.106
Asian	1	102	2.69	1.04-6.97	0.042	0.084	-	-	
Mixed	2	373	1.08	0.61-1.90	0.795	1.59	3.28	69.51	
**BB *vs* AB+AA**									0.729
Asian	1	102	1.00	0.02-51.36	1.000	2	-	-	
Mixed	2	373	0.46	0.05-3.91	0.473	0.946	0.18	0.00	

K= Total number of studies, N= Total number of participants, ES= Effect Size, CI= Confidence Intervals, A/B allele= G/A allele, * $P_{heterogeneity}$ <0.05, ** $P_{heterogeneity}$ <0.01, *** $P_{heterogeneity}$ <0.001, p_c = p-value after Bonferroni correction (P×2 ethnic groups), 'a' p-value for comparison between subgroups
